# A Smartphone App to Support Adherence to Inhaled Corticosteroids in Young Adults With Asthma: Multi-Methods Feasibility Study

**DOI:** 10.2196/28784

**Published:** 2021-09-01

**Authors:** Jane Murphy, Jenny McSharry, Lisa Hynes, Gerard J Molloy

**Affiliations:** 1 School of Psychology National University of Ireland, Galway Galway Ireland; 2 Croí, The West of Ireland Cardiac Foundation Galway Ireland

**Keywords:** asthma, young adult, medication adherence, self-management, mobile applications, mHealth, intervention, usability, acceptability, feasibility, multi-methods, mobile phone

## Abstract

**Background:**

Young adults with asthma often report low adherence to inhaled corticosteroids (ICS), leading to uncontrolled symptoms and poor disease outcomes. Technology-enabled digital supports such as mobile health (mHealth) asthma smartphone apps have the potential to support adherence to ICS and asthma self-management. There is a need for feasibility studies to determine the usability, acceptability, and feasibility of these interventions. In addition, it is essential to determine the feasibility of recruiting and retaining young adults to plan future efficacy and effectiveness trials and therefore, establish evidence-based asthma apps.

**Objective:**

This study aimed to determine (1) the feasibility of recruiting and retaining young adults to a trial and (2) the usability, acceptability, and feasibility of using the AsthmaMD app to support adherence to ICS in a population of young adults living with asthma.

**Methods:**

A multi-methods feasibility study was conducted. Young adults aged 18-30 years with asthma and current prescription for ICS were eligible and invited to take part through a university circular email, social media, and general practice sites. Participation involved completing a baseline self-report questionnaire, downloading and using the AsthmaMD app for 2 weeks, and completing the follow-up assessment, including self-report and open-ended questions about participants’ experience of using the app. Primary outcomes included participant recruitment and retention and the usability, acceptability, and feasibility of using AsthmaMD. Quantitative self-report data were analyzed using descriptive statistics, and qualitative open-ended data were analyzed using inductive reflexive thematic analysis.

**Results:**

A total of 122 young adults (females, n=101, 82.8%) with a mean age of 24.4 (SD 3.8) years were recruited and they completed baseline measures. Of the 122 young adults, 59 (48.4%) completed the study. The AsthmaMD app received a mean score of 63.1/100 (SD 20.1) on the System Usability Scale (ie, a standardized measure of usability for technology-based apps), and an overall user satisfaction score of 5.8/10 (SD 2.2). Of the 59 participants who completed the study, 49 (83%) participants used the app ≥1 day per week. Two main themes were identified in the qualitative analysis of user experiences: (1) learning how to use the app to suit the individual and (2) benefits and relevance of using the app.

**Conclusions:**

The findings from this study indicate that it is feasible to recruit and retain young adults to examine efficacy and effectiveness in a future trial and that young adults living with asthma may find AsthmaMD to be usable, acceptable, and feasible to support adherence to ICS. Our findings also identified opportunities to further optimize the usability of AsthmaMD and similar apps. Based on our findings, we recommend providing more accessible information on how to use the app and replacing medical terminology with simplified language within the app to improve usability.

**Trial Registration:**

ISRCTN Registry ISRCTN11295269; https://www.isrctn.com/ISRCTN11295269

## Introduction

### Background

Asthma is a major global health concern with increasing prevalence currently affecting up to 339 million people [1]. More than 60% of adults living with asthma have uncontrolled symptoms [2,3], leading to lower quality of life and higher productivity loss, health care utilization, and asthma mortality [4-7]. Asthma can be effectively controlled through patient engagement in self-management behaviors such as symptom monitoring, avoiding triggers, and adherence to appropriate treatments [8]. According to the Global Initiative for Asthma guidelines [9], adherence to inhaled corticosteroids (ICS), which are considered the most effective long-term asthma control medication [10-13], is essential for the effective management of asthma*.* Adherence to ICS has been consistently associated with asthma control, improved lung function, reduced symptom exacerbations, and thereby reduced burden of asthma on health care systems [14-17]. However, low adherence remains a major barrier to optimal asthma control, most notably in younger populations [18,19].

A recent review estimated that only 28% of young adults may be fully adherent to their ICS [20]. Adherence to ICS in young adults is further complicated as they become responsible for their own asthma self-management, with parents or caregivers potentially having less involvement in these tasks [21]. This alone constitutes a vulnerable transitional period for young adults [22]. Additionally, during this period, young adults often enter a psychological development phase, coined as emerging adulthood [23,24]. Emerging adulthood has been proposed as a distinct stage of development between adolescence and adulthood from approximately the age of 18 years to 29 years, where individuals typically experience greater autonomy, explore opportunities in education, work, residence, and relationships [23,24], and engage in more health risk behaviors [25-28]. Asthma self-management is affected by this unstable development period. Furthermore, engaging young adults in self-management research often proves challenging in asthma [29] and other chronic conditions [30,31]. Therefore, this population requires attention and there is a clear need to establish appropriate supports to improve self-management, especially adherence to ICS, at this challenging point in the lifespan.

Smartphones provide an existing intervention platform for young adults, given their near universal ownership and high daily use in this population [32]. Although growth in their ownership has not always been equal within or between countries, these devices are now widely used across socioeconomic groups, with young adults being the most likely cohort to own a smartphone in both high and low- and middle-income countries [32]. Moreover, there has been an exponential growth of commercially available mobile health (mHealth) apps, including asthma self-management apps, some of which have demonstrated potential to improve adherence and disease control [33,34]. A recent systematic review [35] identified AsthmaMD as one of the currently available asthma apps with the highest number of evidence-based behavior change techniques (BCTs; n=10) [36] and highest mobile app rating score (score 4.23/5)*.* Additionally, recent qualitative work has explored the technology preferences of young adults with asthma to support adherence to ICS (J Murphy, MSc, under review, November 2020). Young adults’ preferred type of technology to support adherence was a smartphone app, and accordingly, an app is the focus of this study. Based on their preferences for individual app features, we selected AsthmaMD as a suitable, freely available asthma app for this population. However, it is not yet known whether young adults consider this app easy-to-use, acceptable, and feasible, which are essential to ensure successful uptake and use [37,38].

### Research Questions

The aim of this study was to assess the feasibility of recruiting and retaining young adults to a trial and the usability, acceptability, and feasibility of using the AsthmaMD app to support adherence to ICS in a population of young adults living with asthma in Ireland. The research questions in this study are as follows:

1. Can participants be recruited to take part in the study? (feasibility of young adult recruitment)
2. Can participants be retained in the study until completion? (feasibility of young adult retention)
3. Do participants find AsthmaMD easy to use? (usability of AsthmaMD)
4. Are participants satisfied with using AsthmaMD to support adherence to ICS and with their overall user experience with the app? (acceptability of AsthmaMD use)
5. Do participants use and would they continue to use AsthmaMD to support adherence to ICS? (feasibility of AsthmaMD use)

Following guidance on conducting feasibility studies [39], the research team established Go/No Go progression criteria to determine if the findings of this study indicated that it would be feasible to examine the efficacy and effectiveness of AsthmaMD in a future randomized controlled trial.

## Methods

### Design

A multi-methods feasibility design [40] with a 2-week follow-up was employed for this study. The intervention duration was based on input from public and patient involvement (PPI) contributors who felt this was an appropriate duration to obtain a sufficient user experience and that their engagement with the app over this period would accurately indicate their long-term use of the app. This 2-week follow-up is consistent with similar feasibility studies of eHealth and mHealth interventions such as apps for medication adherence [41] and self-management [42], including those for asthma specifically [43,44]. Additionally, a recent app retention report found that the largest reduction in health-related and medical app users occurs from day 1 to day 3, while the lowest reduction occurs from day 14 to day 30 [45]. This indicates that a significant proportion of users who will not use these apps in the long term will stop doing so within 3 days and therefore, are likely to be captured within a 2-week period. All data were collected online via LimeSurvey (version 3.19) [46] from September to December 2020. Participants provided informed consent electronically. Participant information sheet and consent form are provided in Multimedia Appendix 1 and Multimedia Appendix 2, respectively. Ethical approval was granted by the relevant University Ethics Committee (reference number: 20-Jan-13) on February 18, 2020.

This study was reported in accordance with all relevant principles of the extended CONSORT (Consolidated Standards of Reporting Trials) checklist of information to include when reporting a pilot/feasibility trial [47], the CONSORT-EHEALTH checklist, for improving and standardizing evaluation reports of web-based and mHealth interventions [48], the Template for Intervention Description and Replication (TIDieR) checklist [49] to ensure completeness of reporting and replicability of the intervention, and the Checklist for Reporting Results of Internet E-Surveys (CHERRIES) [50]. The completed checklists are shown in Multimedia Appendix 3, Multimedia Appendix 4, Multimedia Appendix 5, and Multimedia Appendix 6, respectively.

### Go/No Go Progression Criteria

In line with relevant guidance [40], the research team identified the key uncertainties that needed to be assessed in relation to the feasibility of using AsthmaMD and examining its efficacy in a future trial in young adults. These key uncertainties were identified from the relevant literature and research team expertise in relevant parameters of feasibility studies. Following discussion, the research team agreed the Go/No Go progression criteria for the study and how these criteria would be interpreted accordingly. Criteria and thresholds were based on recommended sample size for feasibility studies [51], attrition rates in similar studies in young adults [41,42,52,53], validated usability scales and cut-off scores [54,55], and previously used measures and definitions of acceptability [56,57] and feasibility of app use [56-60]. The rationale for the selected thresholds is further outlined in Multimedia Appendix 7. The progression criteria and thresholds for each are presented in Table S1 of Multimedia Appendix 8. The scoring key was devised based on a recent precedent [58]. The scoring key was as follows:

1. If all criteria are rated as green, the trial to test the effectiveness of the app can immediately proceed.
2. If some criteria are partially met and rated as amber, the trial of the app can proceed once relevant criteria have been reviewed and a plan to make relevant amendments has been agreed by the research team.
3. If one criterion is not met and rated as red, the trial can only proceed if (1) no more than 2 criteria are rated red and (2) a plan to make the required amendments has been agreed by the research team.

### PPI

A total of 4 (2 females) young adult PPI contributors in the age range of 19-27 years, living with asthma, and prescribed ICS were recruited through social media and advertisements in the university campus. They were invited to attend 1 meeting lasting approximately for 1.5 hours. Each contributor was offered a €38 (US $1=€0.85) retail voucher for their contributions. This value was based on INVOLVE guidelines [61], the United Kingdom’s national advisory group to support public involvement in the National Health Service research. The researcher held 2 meetings, each with 2 young adult contributors. The researcher presented the proposed research and contributors were asked to review all study documents individually, including the study information sheet, consent form, invitation letter from a general practitioner (GP), baseline and follow-up measures, and then to discuss their reviews with the group. Specifically, the researcher asked the contributors to review all the documents for clarity and relevance from a lived experience perspective. The appropriate study follow-up duration and recruitment strategies were then discussed among the group*.* According to a recent PPI Involvement Matrix [62], the contributors fulfilled the cothinker and advisor role at the implementation stage of this research. Appropriate and feasible amendments were made to the study design and documents following each meeting.

Examples of PPI input into the study design and amendments include (1) the 2-week follow-up duration based on the reasons outlined in the design section, (2) using a different font such as Times New Roman in all study documents, as this is often the required font for college-related submissions and can remind young adults of academic work, which may prevent engagement with the study information, (3) providing a description and common examples of oral corticosteroids to increase clarity when asking participants if they have been treated with these in the past year, and (4) increasing the use of terms such as “novel,” “innovative,” and “unique” when describing AsthmaMD as a potential method to support adherence, stimulate young adult interest, and emphasize the app as an alternative to traditional clinical interventions.

### Participants and Recruitment

Eligible young adults were aged 18-30 years, with a self-reported asthma diagnosis and currently prescribed a form of ICS. Young adults were defined as adults aged 18-30 years to include the emerging adulthood age range of 18-29 years [23,24] and the range of definitions across the health care transition literature [63,64]. This definition has also been used previously in a similar context [65]. Participants were not compensated or offered any incentive to take part in the study. They were invited to take part through an email sent from a weekly university circular to all registered students. The study was also advertised via social media from several accounts including the study’s own Facebook, Twitter, and Instagram accounts, the Asthma Society of Ireland’s Twitter and Facebook, and a range of other community and health-based social media accounts. Additionally, the 4 PPI study contributors shared the study advertisement via their social media. Four GPs in the Galway region also supported study recruitment. The GPs identified all eligible patients on their registers, following a search of 18-30-year-old adults who were coded with an asthma diagnosis and prescription for ICS. Collectively, they identified 85 eligible patients who were posted a study invitation. As an incentive to support recruitment, GPs were offered an Irish Medical Council–eligible audit template of clinically relevant Global Initiative for Asthma guidelines [9] that was conducive to fulfilling their annual audit requirements.

### Sample Size

A minimum of 59 participants were required to complete this study. This target was based on a commonly applied recommendation from the literature, which states that if a problem with 5% probability exists in a potential study participant, then it should be identified in a sample of 59 participants [51]. Based on attrition rates in similar feasibility studies with a 2-week follow-up [41,42,52] and a recent pilot study of an asthma self-management app in a population of young people [53], we anticipated a 25% rate of attrition in this study. To allow for this, we aimed to recruit a minimum of 74 participants for this study.

### Outcome Measures

Primary outcomes were the feasibility of participant recruitment and retention, and the usability, acceptability, and feasibility of AsthmaMD. Additional measures were used to collect information on demographics, asthma characteristics, adherence to ICS, asthma control, and smartphone and app use. All baseline and follow-up measures are included in Multimedia Appendix 9 and Multimedia Appendix 10, respectively. Baseline demographic and asthma measures included age, gender, education, ethnicity, eligibility for free or reduced-rate medical treatment and GP services, number of years since asthma diagnosis, emergency department visits, hospital admissions, and treatment with oral steroids in the past year. Adherence to ICS was measured at baseline and at follow-up by using the Medication Adherence Report Scale for Asthma (MARS-A) [66], a 10-item self-report measure. Responses ranged from “(1) Always” to “(5) Never,” where higher scores indicate better adherence. Responses were summed to yield a total score from 10 to 50. The MARS-A demonstrated reliability and construct and convergent validity [67,68]. Asthma control was measured at baseline and at follow-up by using the Asthma Control Test (ACT) [69]. The ACT is a 5-item questionnaire, to which responses are rated on a 5-point scale and summed to provide a total score ranging from 5 to 25, with higher scores representing better asthma control. An ACT score ≤19 indicates uncontrolled asthma [69]. The ACT is the most validated composite measure of asthma control [70-72]. Additionally, information on smartphone and app use were collected at baseline, including number of years using a smartphone, hours per day using apps, ever used an asthma app, and current use of and awareness of an asthma app.

### Feasibility of Participant Recruitment

Feasibility of participant recruitment was determined by the number of participants recruited, that is, the number of participants who provided consent and completed baseline measures. The source of recruitment was also recorded by asking participants where they heard about the study.

### Feasibility of Participant Retention

Feasibility of participant retention was determined by the number of participants who completed the follow-up assessment, that is, those who completed the study.

### Usability of AsthmaMD

The perceived usability of AsthmaMD was determined using the System Usability Scale (SUS) [54] at follow-up. The SUS consists of 10 items to which responses are made on a 5-point scale ranging from “(1) strongly disagree” to “(5) strongly agree”. Scoring of the SUS involves recoding responses. Reponses to the odd-numbered items (1, 3, 5, 7, and 9) are subtracted by 1, and responses to the even-numbered items (2, 4, 6, 8, and 10) are subtracted from 5. These recoded responses are then summed, and the sum is multiplied by 2.5 to generate a composite SUS score ranging from 0 to 100, with higher scores indicating greater usability. A score >68 is considered above average [55]. The SUS has demonstrated reliability and convergent and discriminant validity across a range of populations [55,73,74]. It is technology agnostic [75] and therefore remains applicable to a range of technologies as they continuously evolve, such as smartphone apps. Participants were also asked if the app was easy to use, which individual app features they used, and which features they found useful.

### Acceptability of AsthmaMD Use

Acceptability of AsthmaMD use was determined at follow-up from participants’ ratings of their overall user satisfaction on a scale of 1-10, whether the app increased adherence awareness and adherence behavior, increased confidence, reduced stress in managing ICS, and if notifications were annoying. Participants’ willingness to recommend the app to another person with asthma and to pay for the app were also measured. These measures are modified from previous feasibility studies of mHealth interventions [56,57], one of which defined acceptability of app use as ≥30% of participants agreeing to similar questions [57].

### Feasibility of AsthmaMD Use

Feasibility of app use was determined at follow-up from the self-reported number of days per week participants used the app and their intention to continue to use the app beyond their participation in the study. Participants’ duration of app use was also measured by the self-reported number of minutes per day they used the app. These are frequently used questions in feasibility studies of mHealth interventions [56-60].

### Open-ended Questions

Participants were asked 5 open-ended questions at follow-up, which aimed to elicit additional feedback and elaboration of their experience with using the app. These questions were primarily adapted from a recent similar study [53] and included what participants liked the most and the least about the app, any difficulties experienced, suggestions for improvements, and any other comments.

### Intervention

AsthmaMD was created by Dr Sam Pejham, a physician and researcher from the University of California, San Francisco (UCSF) Medical School Clinical Faculty. The app was awarded the UCSF Collaborative Research Network grant, which is a primary care practice–based research network grant. A recent review identified the following BCTs in AsthmaMD: (1) information about health consequences, (2) salience of consequences, (3) information about others’ approval, (4) instruction on how to perform a behavior, (5) demonstration of the behavior, (6) self-monitoring of behavior, (7) self-monitoring of outcome(s) of behavior, (8) feedback on behavior, (9) feedback on outcome(s) of behavior, and (10) prompt/cues*.* The first author (JM) also screened and coded AsthmaMD for the presence of BCTs by using the BCT taxonomy (BCTTv1) [36]. Consistent with Ramsey et al [35], JM identified the presence of these 10 BCTs, with the addition of credible source in each video tutorial as Dr Pejham delivers the information and either speaks in favor of the relevant self-management behavior or using AsthmaMD to support these behaviors. The app was available to download for free from Google Play Store (Android) and App Store (iPhone). Screenshots of the AsthmaMD user interface and its individual features can be seen in Multimedia Appendix 11.

AsthmaMD allows users to log their peak expiratory flow meter readings, which can indicate response to treatment and triggers for worsening symptoms or determine a baseline for action plans [9]. In addition to peak expiratory flow readings, the app allows users to log their symptoms, triggers, medications, and other notes in the form of a diary and share these data with their physicians or other persons. It provides a line chart of peak expiratory flow meter readings and symptoms, indicating the severity of each, allows users to create customized reminders to take their medication, and guides users through their asthma action plan. Finally, the app provides video tutorials on using your peak flow meter, understanding asthma and asthma medication, and using AsthmaMD.

### Procedure

The procedure involved a baseline and follow-up web-based questionnaire administered by LimeSurvey (version 3.19) [46]. Participants were asked to download AsthmaMD at the end of the baseline questionnaire. They were not instructed on how, when, how often, or how long to use AsthmaMD. It was decided to leave the frequency and duration of use to the users’ discretion as people mainly use these technologies independently and autonomously [38]; thus, this may provide a more accurate indication of how participants would use the app in their daily life after study participation, that is, their long-term use of the app. Participants could tailor the app to their own personal regime at their discretion, for example, by entering the name and dosage of their medications at any point during the 2 weeks. No other physical or informational materials were provided to participants as part of the intervention. Participants were encouraged to make themselves familiar with the app’s data privacy policy before downloading the app. If participants were interested in completing the follow-up questionnaire, they were asked to provide their email address and they received an email with the link to the follow-up study 2 weeks later from the researcher, a PhD candidate in health psychology. If these participants had not completed the follow-up questionnaire within 3 working days, they were sent a total of 3 successive reminder emails every 3 working days.

### Data Analysis

Quantitative analysis was performed using SPSS software (version 26, IBM Corp) [76]. Descriptive statistics were used to analyze baseline data and primary study outcomes. Independent two-tailed *t* tests and *χ*^2^ tests of association were used to examine the differences between participants who completed the study and those who did not, that is, those lost to follow-up. Cronbach α was conducted to examine the internal consistency of the multi-item scales employed.

Qualitative analysis was performed using NVivo software (version 12, QSR International) [77]. An inductive, reflexive thematic analysis [78,79] was conducted on participant responses to open-ended questions in the follow-up assessment. The analysis followed the 6 stages of Braun and Clarke’s [78] proposed guidance: (1) familiarizing oneself with the data, (2) generating initial codes, (3) searching for themes, (4) reviewing themes, (5) defining and naming themes, and (6) producing the report with illustrative quotes. The first author engaged in data immersion by repeatedly reading and coding all data. Initial codes were collated into potential themes relevant to the complete data set. Analysis was iterative with codes, themes, and subthemes refined throughout.

## Results

### Baseline Measures

Table 1 presents the information collected at baseline. The mean age of the sample was 24.4 (SD 3.8) years, and the majority were females of White Irish ethnicity who had at least tertiary or higher-level education and were living with asthma for at least 10 years. On average, the sample reported low adherence to ICS and uncontrolled asthma at baseline and follow-up assessments. The majority were using a smartphone for 5-10 years, they spent at least 3 hours per day using smartphone apps, had not previously and were not currently using an asthma app, and were unaware of asthma apps. Table 1 also provides a comparison of the baseline measures between participants who completed the study and those who did not. Participants who completed the study were significantly older, and a significantly greater proportion had postgraduate education and were aware of an asthma app in comparison to those who did not complete the study. No other significant differences in baseline measures existed between those who did and did not complete the study. Cronbach α for the MARS-A at baseline (α=.83) and follow-up (α=.83), for the ACT at baseline (α=.82) and follow-up (α=.83) and for the SUS at follow-up (α=.92) were all >.70 [80], demonstrating valid internal consistency for all scales in this study.

**Table 1:** Baseline measures according to study completion (N=122).

Variable				Participants who completed baseline (N=122)		Participants who completed baseline and follow-up (n=59)		Participants who completed baseline only (n=63)		*P* value^a^	
**Demographics**											
	Age (years), mean (SD)			24.4 (3.8)		25.1 (3.8)		23.6 (3.6)		.03	
	**Gender, n (%)**										.38
		Female	101 (82.8)		47 (80)		54 (86)				
		Male	21 (17.2)		12 (20)		9 (14)				
	**Education, n (%)**										.04
		Second level	18 (14.8)		4 (7)		14 (22)				
		Tertiary/higher level	63 (51.6)		31 (52)		32 (51)				
		Postgraduate	41 (33.6)		24 (41)		17 (27)				
	**Ethnicity, n (%)**										.49
		White Irish	99 (81.1)		51 (86)		48 (76)				
		Other White background	13 (10.7)		3 (5)		10 (16)				
		Black/Black Irish	2 (1.6)		1 (2)		1 (2)				
		Asian/Asian Irish	3 (2.5)		2 (3)		1 (2)				
		Mixed	3 (2.5)		1 (2)		2 (3)				
		Other	2 (1.6)		1 (2)		1 (2)				
	Medical card holder, Yes, n (%)			25 (20.5)		8 (14)		17 (27)		.07	
	General practitioner visit card holder, Yes, n (%)			10 (8.2)		2 (3)		8 (13)		.06	
	**Recruitment source, n (%)**										.08
		Facebook	29 (23.8)		15 (25)		14 (22)				
		Twitter	23 (18.9)		10 (17)		13 (21)				
		Instagram	22 (18.0)		9 (15)		13 (21)				
		University student mailer	23 (18.9)		7 (12)		16 (25)				
		Word of mouth	23 (18.9)		17 (29)		6 (9)				
		General practitioner/nurse	2 (1.6)		1 (2)		1 (2)				
**Asthma characteristics**											
	**Years since diagnosis, n (%)**										.14
		<1 y	1 (0.8)		0 (0)		1 (2)				
		1-2 y	9 (7.4)		5 (8)		4 (6)				
		2-5 y	14 (11.5)		8 (14)		6 (10)				
		5-10 y	15 (12.3)		8 (14)		7 (11)				
		10+ y	76 (62.3)		38 (64)		38 (60)				
		Don’t know	7 (5.7)		0 (0)		7 (11)				
	Visited emergency department in the last year, Yes, n (%)			13 (10.7)		8 (14)		5 (8)		.32	
	Hospital admission in last year, Yes, n (%)			8 (6.6)		5 (9)		3 (5)		.41	
	Prescribed oral steroids in last year, Yes, n (%)			46 (37.7)		26 (44)		20 (32)		.16	
	**Asthma control, n (%)**										.22
		Uncontrolled asthma: Asthma Control Test score ≤19	64 (52.5)		28 (47)		36 (57)				
		Controlled asthma: Asthma Control Test score >19	58 (47.5)		31 (53)		27 (43)				
	Total Asthma Control Test score, mean (SD), range			19.0 (4.2), 7-25		19.2 (4.4), 7-25		18.8 (4.0), 9-25		.59	
	Adherence, Total Medication Adherence Report Scale for Asthma, mean (SD), range			33.3 (8.6), 15-50		33.4 (8.3), 18-50		33.1 (8.9), 15-50		.85	
**Smartphone and app use**											
	**Years using smartphone, n (%)**										.06
		3-5 y	2 (1.6)		1 (2)		1 (2)				
		5-10 y	67 (54.9)		31 (52)		46 (73)				
		10+ y	39 (32.0)		27 (46)		16 (25)				
	**Hours per day using apps, n (%)**										.96
		1-3 h	38 (31.1)		22 (37)		22 (35)				
		3-5 h	47 (38.5)		26 (44)		29 (46)				
		5+ h	23 (18.9)		11 (19)		12 (19)				
	Ever used asthma app, Yes, n (%)			4 (3.3)		2 (3)		2 (3)		.95	
	Currently using asthma app, Yes, n (%)			5 (4.1)		4 (7)		1 (2)		.15	
	Aware of any asthma app, Yes, n (%)			10 (8.2)		8 (14)		2 (3)		.04	

^a^*P* value refers to independent two-sided *t* tests or *χ*^2^ tests of association.

### Primary Outcomes

#### Participant Recruitment

A total of 122 participants were recruited to this study and they completed baseline measures from September to December 2020. In relation to where the participants heard about the study, Facebook was the most common source, followed by Twitter, university student mailer, word of mouth, Instagram, and GP or nurse.

#### Participant Retention

Of the 122 participants who completed the baseline measures, 59 (48.4%) completed the follow-up assessment, that is, were retained until study completion. Information regarding participant flow throughout the study is shown in Multimedia Appendix 12. Of the participants who completed the study, word of mouth was the most common recruitment source, followed by Facebook, Twitter, Instagram, university student mailer, and GP or nurse.

#### Usability of AsthmaMD

The mean SUS score for AsthmaMD was 63.1 (SD 20.1). A score >68 indicates average usability. A total of 25 (46%) participants “agreed/strongly agreed” that the app was easy to use. Table 2 presents the proportion of participants who used individual AsthmaMD features and who found these useful. The feature that was most used and most frequently reported as useful was the symptom log. The feature that was least used and least frequently reported as useful was the forced expiratory volume-1 log.

**Table 2:** AsthmaMD features, behavior change techniques present, and proportion of participants who used and found each feature useful (n=59).^a^

App feature	Behavior change technique	Participants who used feature, n (%)	Participants who found feature useful, n (%)
Symptom log	Self-monitoring of behavior Self-monitoring of behavior outcome(s)	41 (70)	30 (73)
Log of medication use	Self-monitoring of behavior	39 (66)	18 (46)
Trigger log	Self-monitoring of behavior	37 (63)	29 (78)
Peak flow log	Self-monitoring of behavior outcome(s)	13 (22)	10 (77)
Oximetry log	Self-monitoring of behavior outcome(s)	3 (5)	2 (67)
Forced expiratory volume-1 log	Self-monitoring of behavior outcome(s)	0 (0)	0 (0)
Notes	Self-monitoring of behavior Self-monitoring of behavior outcome(s)	5 (9)	2 (40)
Diary	Feedback on behavior	26 (44)	17 (65)
Line chart	Feedback on behavior outcome	26 (44)	17 (65)
Send report to physician/other	Information about other’s approval	3 (5)	2 (67)
Reminders	Prompts/cues	23 (39)	18 (78)
Action plan	Instruction on behavior performance	12 (20)	7 (58)
Video tutorial on using your peak flow meter	Instruction on behavior performance Demonstration of behavior Credible source	3 (5)	2 (67)
Video tutorial on understanding asthma	Information about health consequences Salience of consequences Credible source	9 (15)	7 (78)
Video tutorial on asthma medication	Information about health consequences Instruction on behavior performance Credible source	6 (10)	5 (83)
Video tutorial on AsthmaMD	Instruction on behavior performance Demonstration of behavior Credible source	5 (9)	3 (60)
AsthmaMD frequently asked questions	Instruction on behavior performance	6 (10)	1 (17)

^a^The number of participants who used each feature and who found each feature useful varied per individual feature.

#### Acceptability of AsthmaMD Use

AsthmaMD received a mean score of 5.8 (SD 2.2) for participants’ overall experience in using the app for managing their adherence to ICS. Of the 59 participants, 34 (58%) participants rated their experience ≥5/10. In total, 27 (46%) participants “agreed/strongly agreed” that the app increased their awareness of their adherence, and 21 (36%) “agreed/strongly agreed” that the app increased their adherence to ICS. In response to whether the app increased their confidence in managing their ICS, 18 (31%) participants “agreed/strongly agreed.” A total of 15 (25%) participants “agreed/strongly agreed” that the app reduced the stress in managing their adherence to ICS, and 25 (42%) “agreed/strongly agreed” that the app notifications did not annoy them. A total of 25 (42%) participants reported that they would recommend the app to another person with asthma and 14 (24%) were “not sure.”

#### Feasibility of AsthmaMD Use

In total, 49 (83%) participants reported using AsthmaMD ≥1 day per week. The mean number of days per week participants reported using the app was 3.2 (SD 1.9). In response to whether participants would continue to use the app after their participation in the study, 16 (27%) said “yes” and 14 (24%) were “not sure.” In relation to number of reported minutes per day participants used the app, 26 (44%) used it for less than 5 minutes, 22 (37%) used it for 5-10 minutes, 7 (12%) used it for 10-15 minutes, and 4 (7%) used it for 15-20 minutes per day.

### Qualitative Findings From Open-ended Questions

Of the 59 participants, 40 (68%) participants responded to the open-ended questions. Two main themes were identified in the data: (1) learning how to use the app to suit the individual and (2) benefits and relevance of using the app.

#### Learning How to Use the App to Suit the Individual

Participants had a range of experiences with learning how to use AsthmaMD and making it personally useful. Some discussed how easy the app was to use on first use, for individual features and overall.

…It was easy to input my personal data and then to track symptoms and triggers.

Participant 47, male, age 25 years, tertiary/higher level education

Other participants felt the app required an investment of time for *“*trial and error” (Participant 23, female, age 30 years, tertiary/higher level education) in the beginning to learn how to use it and subsequently to understand how it could be useful. This initial investment was worthwhile for some who progressed in using the app thereafter and found individual features useful that they may otherwise not have used.

…It took me a while to fully understand the proper function of the app…to realize what the action plan was and what it was for…Once I understood it made more sense to me why I would use an app like this…I will be looking into getting an action plan in place with my GP which I wouldn’t have thought of before downloading the app.

Participant 39, male, age 27 years, postgraduate education

However, despite this time investment, some participants continued to find the app difficult to use. Two factors contributed to this experience. First, participants commented on the unnoticeable location of features such as the AsthmaMD app video tutorial, which may have enabled participants to more efficiently and easily learn how to use the app if it had been more apparent to the user.

…I couldn’t see where to access videos/tutorials to help with using the app which would’ve been useful.

Participant 14, female, age 20 years, second-level education

Participants suggested solutions to this, including relocating the information and features on how to use AsthmaMD that already exist within the app to a more obvious location that is easier for users to find and adding more information on how to use individual features, immediately presenting this to users on their first use of the app. Specifically, suggestions included adding a help section within each feature on how and why to use it.

…Help sections within each section: eg, when I click on “action plan” it would be useful if there was a link to help with that - Other apps have a question mark at the top which when you click it tells you about the section, eg, directions on how to set it up and when and why it would be helpful…I would also include information about what the action plan is and how to go about getting in with the GP”

Participant 11, female, age 29 years, postgraduate education

Second, participants felt there was too much medical jargon used in the app. They felt the app assumed user knowledge of terminology, which they did not have, thereby making it difficult to understand.

…I wasn’t familiar with some of the terms…the app just presumes you know things.

Participant 57, female, age 21 years, tertiary/higher level education

#### Benefits and Relevance of Using the App

This theme includes 2 subthemes: (1) prompt to action and (2) useful for me or for others.

##### Prompt to Action

Participants described how their use of the app resulted in increased awareness of their asthma self-management, which often resulted in increased self-management behavior, including improved adherence to ICS and seeking a GP consultation to track peak flow and devise an asthma action plan.

…this app has highlighted areas of my asthma that I wasn’t aware of. I have booked in to my GP to get some answers and to be able to track the peak flow.

Participant 41, female, age 26 years, tertiary/higher level education

One participant discussed how the app identified their asthma as suboptimally controlled, which prior to their use of this app they perceived as well-controlled. This objective indication of asthma control may have otherwise remained unknown to the participant who now intends to improve their self-management as a result.

…Reminded me about some of the measures to control your asthma. I thought I managed mine well, but the app told me it wasn’t so I have to improve on it and I definitely will, due to the app!

Participant 62, male, age 30 years, tertiary/higher level education

In particular, the reminder to take ICS was discussed as a useful feature to overcome forgetfulness, which was noted as a barrier to taking ICS, and thereby improve adherence to this medication. However, 1 participant felt the regular reminders unnecessarily increased their awareness of taking their ICS and made the behavior feel more effortful.

…It made taking my inhaler into a chore. I’m aware that it is a chore but the constant notifications annoyed me.

Participant 54, male, age 19 years, second-level education

##### Useful for Me or for Others

Participants discussed the individual features they found and would find especially useful based on their personal self-management needs and preferences at different times.

…This would be very handy when I need to track my peak flow.

Participant 34, female, age 28 years, tertiary/higher level education

…I used this app to track my peak flow, I take my inhaler properly every day so did not need it to help me with this.

Participant 50, female, age 28 years, postgraduate education

Others expressed how they did not find the app particularly useful owing to a lack of perceived asthma severity, already having a daily routine for taking ICS or already using a reminder on their phone, and perceiving no need to use additional technology features.

…the app would be good for someone that isn’t compliant with their medications however I am so used to taking it now I don’t need it.

Participant 12, female, age 26 years, tertiary/higher level education

A common perception among these participants was that although the app may not be useful for them on a personal level, they felt it would be useful for other young adults living with asthma such as those with low adherence to ICS, more severe or frequent symptoms, or those newly diagnosed with the condition.

…this might be more useful for moderate/severe asthma patients who are struggling to determine their disease pattern. I can see the benefit of the app for this type of patient. It seems less useful for controlled asthma, except for the reminders.

Participant 21, female, age 30 years, postgraduate education

Participants proposed suggestions to increase the personal relevance of the app by adding all ICS treatments to the medication list within the app, adding options to set reminders for additional self-management behaviors such as logging symptoms and visually presenting the relationship between users’ data on symptoms and medication use in a chart.

…a flow chart of your symptoms and it’s correlation with medication use…that would be useful.

Participant 2, female, age 24 years, postgraduate education

## Discussion

### Principal Findings

The findings from this study indicate that the AsthmaMD app is usable, acceptable, and feasible as defined by the study progression criteria, to support adherence to ICS in a population of young adults living with asthma, and that it appears feasible to recruit and retain young adults to examine its effectiveness in a prospective randomized controlled trial. Table S2 of Multimedia Appendix 13 presents the outcome of each Go/No Go progression criterion. All criteria were fully met (Green: Proceed), except for the usability of AsthmaMD, which was partially met (Amber: Amend). Based on our findings, we propose 3 strategies to improve the app’s usability.

First, we propose relocating the AsthmaMD app video tutorial to the main app home screen and sending a notification that directs users to this tutorial when they first download and open the app. Second, we suggest adding a help section within each feature that explains how to use it and the benefits of use. Third, we propose removing all medical terminology and simplifying the language used. A recent theory-based reframing approach to address asthma perceptions and ICS treatment beliefs offers an alternative from the traditional medical explanation [81]. This approach describes asthma as a condition in which the lungs are “out of balance” and “overreact” in response to triggers. ICS are described as “natural helpers” that “top up” natural steroids in the body to prevent overreactions and restore balance in the lungs. Initial acceptability has been demonstrated in young adults who perceived the approach as coherent and easy to understand [81]. In particular, they appreciated its use of nonmedical jargon and felt the language presented the information in a more positive manner [81]. Perhaps AsthmaMD could employ the language used in this reframing approach to increase its usability.

The progression criteria for the feasibility of participant recruitment and retention were fully met; however, the apparent rate of attrition (63/122, 51.6%) was twice the anticipated rate (31/122, 25.4%). However, it must be noted that only 63.9% (78/122) of the baseline sample provided their email address to receive and complete the follow-up assessment. Therefore, it may be argued that it was only possible for this proportion of participants to complete the study, meaning the study had an attrition rate of 24% (19/78). After completing the baseline questionnaire, the following page thanked participants for taking part and asked those who wished to complete the follow-up assessment to follow a link and provide their email address. This is a necessary ethical measure to maintain confidentiality by ensuring email addresses are not linked to any other data provided by participants. Although participants may have chosen not to provide their email address at this point, it is likely that some did not finish reading this page and therefore unintentionally did not provide their email address to take part in the follow-up. Asking participants to provide their email address at an earlier point in the baseline questionnaire could help overcome this issue in future research.

Nonetheless, we recommend that future trials being conducted with a similar design factor in an attrition rate of at least 24% over 2 weeks. It is important to recognize that attrition rates are likely to be lower where there is direct participant contact as this may enhance engagement and commitment to the study through the establishment of a researcher-participant relationship [82]. The importance of this personal connection is reflected in the findings with word of mouth being the most effective way of retaining participants. The research team may increase the use of word of mouth by attending and providing study information at university lectures, sports club training events, fundraising events, and Asthma in the Pharmacy Days (events at which an asthma nurse offers patient education on asthma in designated pharmacies nationwide). Word of mouth was followed closely by Facebook as an effective strategy of recruiting and retaining participants. Therefore, we also recommend that future trials advertise the study on all existing Facebook accounts in addition to other social media platforms of the organizations supporting recruitment. Regardless of the organization’s most active platform, it appears that study advertisements on Facebook engages young adults most likely to complete the study.

To determine the feasibility of AsthmaMD use, the frequency of app use during the study period was considered. This was measured by the self-reported number of days per week that participants used the app. The research team selected using the app 1 day per week or more as the “Green: Proceed” threshold for this criterion. This relatively infrequent use in certain contexts may raise questions about the potential potency of this as a behavior change support for self-management of this chronic condition. However, asthma has a symptomatic/asymptomatic nature and varies over time often due to patient adherence, physical activity, allergen or irritant exposure, seasonal changes, or viral respiratory infections [9]. Therefore, frequency of app use will likely vary depending on the users’ needs and preferences at different times. It may be relevant to gauge the use and usefulness of AsthmaMD and similar apps in line with these patient needs such as reducing asthma symptoms for example. This is consistent with recent findings indicating that patients with asthma may keep a self-management app on their phone despite infrequently using it, in order to potentially use it when needed, to manage increasing symptoms [83].

This is line in with the concept of effective engagement with digital behavior change interventions, defined as adequate engagement with the intervention to achieve intended outcomes [84]. This concept proposes that intervention use alone does not determine health behavior outcomes as these may be influenced by additional factors such as motivation and self-regulation skills. Additionally, users may value alternative outcomes to those intended by the developers. Effective engagement is defined based on the aim of the specific intervention and must be identified within the context of that intervention. Evidence of effective engagement has recently been found in the context of an app for medication adherence in adolescents with asthma [85]. Overall app use was not associated with a difference in adherence; however, use of specific features such as the health care provider chat significantly increased adherence behavior. Therefore, if the efficacy of AsthmaMD is established in future trials, it may be more valuable to identify and encourage effective engagement as opposed to simply more engagement with the app.

The jobs-to-be-done theory [86] provides a similar potential theoretical explanation of this. This framework of consumer action proposes that people use a product or service because it meets a need to get a job done, not for the sake of the product or service itself [87]. Patients may want to reduce symptoms when they occur and may use an asthma app for this purpose. Success is determined by getting the job done [87]. Therefore, when symptoms are eliminated, patient users may feel the app has served its purpose and not perceive a need to continue app use. Although health care providers and behavioral scientists may regard constant patient engagement with self-management supports as ideal, this may not be reasonable or necessary. This threshold of using AsthmaMD at least 1 day per week was exceeded as participants used the app, on average, 3 days per week. In total, 51% (30/59) of the participants reported that they would or were unsure if they would continue to use the app. Improving its usability through the above suggestions may increase intent to continue using AsthmaMD beyond the study.

Additionally, it must be noted that the qualitative findings indicate a common perception that participants feel the app may not be personally useful but would be for others with asthma. This may be due to a lack of perceived asthma severity and ICS necessity beliefs, which have consistently predicted self-management behavior and, specifically, adherence to ICS [88-91]. Furthermore, this perceived personal relevance has been found to influence uptake and engagement with a range of eHealth and mHealth interventions [92] and asthma apps [93]. Therefore, asthma and ICS treatment beliefs may need to be addressed to increase the personal relevance and therefore, use of these interventions to improve suboptimal adherence and self-management in these participants. Horne’s reframing approach [81] aims to modify these perceptions. Given its initial acceptability employing this approach within AsthmaMD may also generate more medically accurate asthma perceptions and ICS beliefs, for example, perceiving asthma as a long-term condition requiring regular ICS medication, and in turn, increasing the personal relevance and therefore, use of the app.

The qualitative findings also indicate a lack of knowledge about certain asthma self-management behaviors such as what is an action plan and the purpose of having this in place. It appears this inadequate knowledge contributed to an initial lack of understanding about how AsthmaMD and specific features could be useful. Perceiving little usefulness or benefits of using the app may prevent user engagement. However, an interest in acquiring this self-management information and engaging in the behavior was apparent. This highlights the need and potential for GPs to assess the knowledge of young adults with asthma and address their needs in consultations such as providing information on asthma action plans and devising the patients’ personal action plans with them. Within these consultations, there is potential for GP advocacy of asthma apps such as AsthmaMD to encourage patient use of these technologies to support self-management. Health care providers have reported significant support for asthma mHealth interventions [94,95] and their professional advocacy of these interventions plays an influential role in patient uptake and use of asthma apps specifically [83] and similar interventions in populations with other chronic conditions [96].

### Strengths and Limitations

This study has several strengths and potential limitations. The sample population had a female majority of White Irish ethnicity and with at least tertiary or higher-level education. Of the participants who completed the study, a significantly higher proportion had postgraduate education and were aware of an asthma app and were significantly older than those who did not. However, although significant, this mean age difference was only 1.5 years, which is unlikely, from a psychological perspective, to be developmentally significant at this point in the lifespan. The female majority partly reflects the gender disparities that exist in asthma. In adulthood, females have increased asthma prevalence (9.8% vs 5.5%) [97], hospitalizations, and mortality [98,99]. Furthermore, females are more likely to be educated about asthma control and management than their male counterparts [100,101]. Therefore, they have a higher asthma burden and appear to take increased action concerning their symptoms, which may partially account for higher female engagement in this study. However, adherence and self-management in all adults with asthma requires attention. Both males and females have reported suboptimal asthma control and adherence to ICS [15,102-105]. Therefore, obtaining user feedback with adequate gender balance is important for developing and assessing suitable asthma apps. Further breakdown of recruitment sources by gender identified word of mouth and Twitter as the highest yielding sources of male participants who completed this study. Future studies should consider using these recruitment strategies to target males specifically, for example, providing study information at male sports club events and advertising via their Twitter accounts. In addition, asthma disproportionately affects racial/ethnic minorities [106] and low socioeconomic groups [107-109]. Therefore, it is essential to engage a diverse sample of young adult users to establish appropriate asthma supports. Snowball recruitment techniques and crowdsourcing may increase diversity in future studies. Additionally, our results are based on a sample of young adults with asthma in a particular geographic and sociocultural context; therefore, further research is needed to support our findings in other contexts.

The high mobility of young adults may have partially accounted for the low recruitment rate from GP letters. In Ireland, 56% of 25-34-year-old adults have received tertiary/higher level education [110]. Young adults often move out of the family home when starting this education and continue to change residence beyond this as they explore career opportunities, cohabitation, or home ownership. Therefore, posting mail to their family home address may not be an effective method of contacting this population. A consequence of this low recruitment from GP was that the sample was primarily recruited through social media and therefore were likely to be more digitally literate and motivated to self-select to participate in self-management research. Future research should explore how best to enroll young adults from GP, including those who are harder to reach.

According to relevant guidance, the design and objectives of feasibility studies should be based on the key uncertainties that exist in relation to the intervention or trial and that a randomized design may not be necessary to address these [40]. Therefore, a nonrandomized design was deemed appropriate for this study. This design is consistent with recent feasibility studies of self-management mHealth interventions for young people with a range of chronic conditions [59,111-115], including asthma [53]. Furthermore, recent randomized feasibility studies of such interventions for young people [116,117] and asthma specifically [44,118] have reported no significant difference in retention rates between intervention and control groups. Therefore, the feasibility of randomizing young adults was not considered a key parameter for this initial investigation in which feasibility of recruitment and retention were deemed more pertinent to focus on at this early stage. However, we recognize that willingness to be randomized is specific to each study context and population and therefore, recommend that future similar studies consider this factor.

Our study was also limited by self-reported app use. We acknowledge that self-report measures often lead to under or overreporting of smartphone use [119-122] and while they can provide useful context [84], they may also lead to biased reporting of engagement in mHealth interventions [123]. However, collecting usage data through the app would have raised substantial ethical and data protection concerns with this study as the research team is not affiliated with the developers of AsthmaMD and so would constitute an additional, external party that would require access to this user data. Therefore, it was not considered worthwhile to pursue this method at this initial stage of app evaluation. However, future studies should consider using these objective measures in combination with self-reported app use that can complement and provide meaningful context to these objective data. We also recommend that future similar studies draw on the Theoretical Framework of Acceptability [124] in designing open-ended questions to capture this concept more extensively.

Finally, the aim of this initial investigation was to determine the usability, acceptability, and feasibility of using the AsthmaMD app as an adherence support. The focus was solely on app use. Accordingly, a 2-week follow-up was deemed appropriate to achieve this aim based on PPI input and supported by similar studies and app retention rates. However, future trials that aim to test the efficacy and effectiveness of AsthmaMD to improve adherence behavior and asthma outcomes should consider a longer follow-up duration that allows time for the clinical effect of ICS to maximize in order to optimally increase asthma control. Although a reduction in inflammation and symptoms can occur within days, it may take ICS several months to reach a plateau and can vary per patient [9-11]. Recent efficacy trials of asthma self-management mHealth interventions have used a 6-month follow-up duration in adults [125,126] and adolescents [127]. This would also provide a representation of longer term app use across this population.

This study also has several strengths, including a near equal proportion of participants with uncontrolled and controlled asthma and a range of adherence to ICS in the baseline and follow-up sample. Therefore, this study obtained user feedback from a sample with symptom control and adherence that is likely representative of the general population of young adults living with asthma. Additionally, PPI was conducted by actively involving the relevant patient group in deciding key aspects of this study design. PPI is valued for several reasons in research. Incorporating the patient lived experience can improve research efficiency by increasing relevance, recruitment, and retention, and benefit meaningful dissemination and implementation [128]. It can also increase the transparency and accountability of research [129]. Therefore, it is likely that PPI enriched the quality and relevance of this study to young adults with asthma. Finally, the Go/No Go progression criteria were developed by a research team with combined expertise on treatment adherence, self-management in young adults, and feasibility studies.

### Conclusion

The long-term effectiveness of asthma apps such as AsthmaMD in young adults remains unknown. Prior to examining app effectiveness is determining app usability, acceptability, and feasibility in the target user population. AsthmaMD was deemed usable, acceptable, and feasible as defined in this study to support adherence to ICS in a cohort of young adults living with asthma. It also appears feasible to recruit and retain young adults for further research studies, which is critical for conducting prospective trials examining efficacy, effectiveness, and cost-effectiveness. Before proceeding further, we recommend improving the usability of AsthmaMD by providing more of and relocating existing information on how to use the app to a more accessible location and replacing medical terminology with the simplified language used in Horne’s [81] reframing approach to asthma and ICS. Nevertheless, this study has demonstrated potential for successful uptake and use of AsthmaMD and similar smartphone apps. Given the ubiquitous use of smartphones, these apps may be a scalable and accessible solution to support adherence to ICS in young adults as they typically experience additional developmental demands throughout this stage of the lifespan. Improving suboptimal adherence to ICS is essential to effective asthma management and to reduce the significant burden of uncontrolled asthma on a personal, social, and economic level.

## Appendix

Multimedia Appendix 1 Participant information sheet.

Multimedia Appendix 2 Study consent form.

Multimedia Appendix 3 CONSORT (Consolidated Standards of Reporting Trials) checklist for reporting a pilot or feasibility trial.

Multimedia Appendix 4 CONSORT-eHEALTH (Consolidated Standards of Reporting Trials-eHealth) checklist V1.6.1.

Multimedia Appendix 5 The Template for Intervention Description and Replication (TIDieR) checklist for intervention description and replication.

Multimedia Appendix 6 CHERRIES (Checklist for Reporting Results of Internet E-Surveys) for this study.

Multimedia Appendix 7 Rationale for Go and No Go progression criteria.

Multimedia Appendix 8 Go and No Go progression criteria.

Multimedia Appendix 9 Baseline questionnaire.

Multimedia Appendix 10 Follow-up questionnaire.

Multimedia Appendix 11 Screenshots of AsthmaMD user interface.

Multimedia Appendix 12 CONSORT (Consolidated Standards of Reporting Trials) 2010 flow diagram.

Multimedia Appendix 13 Outcomes of Go and No Go progression criteria.

## Abbreviations

ACT: Asthma Control Test
BCT: behavior change technique
CONSORT: Consolidated Standards of Reporting Trials
GP: general practitioner
ICS: inhaled corticosteroids
MARS-A: Medication Adherence Report Scale for Asthma
mHealth: mobile health
PPI: public and patient involvement
SUS: System Usability Scale
UCSF: University of California, San Francisco
