# Supplementary material for: A Smartphone App to Support Adherence to Inhaled Corticosteroids in Young Adults With Asthma: Multi-Methods Feasibility Study
Source: JMIR Form Res. 2021 Sep 1;5(9):e28784. doi: 10.2196/28784 (PMC8444040; doi:10.2196/28784)
Supplement: Multimedia Appendix 1 [file formative_v5i9e28784_app1.pdf]

A smartphone application to support adherence to preventer inhalers in young adults with asthma: Participant information sheet

You are invited to take part in a research study. Before you decide, it is important that you understand why the research is being done and what it will involve. This participant information sheet tells you about the purpose, risks and benefits of this research study. If you agree to take part, we will ask you to provide consent. If there is anything that you are not clear about, we will be happy to explain it to you. You should only consent to participate in this research study when you feel you understand what is being asked of you, and you have had enough time to think about your decision.

Purpose of the Study

The purpose of this study is to assess the use of a smartphone app relating to preventer inhaler use, among young adults with asthma.

Taking Part – What it Involves

Do I have to take part?

If you decide to take part, you will be asked to provide electronic consent in the following section. You are free to withdraw at any time and without giving a reason. A decision to withdraw, or a decision not to take part, will not affect your rights in any way.

What will happen to me if I take part?

If you decide to take part, you will be asked to:

- Complete a questionnaire, which will take approximately 4 minutes to complete. This will ask about your demographics, asthma characteristics, smartphone experience and app use.
- Download the AsthmaMD app and use the app as you wish for 2 weeks.
- Complete a follow-up questionnaire, which will take approximately 10 minutes to complete. This will ask about your experience of using the app.

Your participation in the follow-up questionnaire will also be completely optional and you are free to withdraw at any time. If you wish to complete the follow-up questionnaire, you will be asked to provide your email address so that the researcher can contact you. Your email address will not be linked to any other information that you provide throughout this study. Furthermore, you will be instructed on how to create a unique personal identifying code, so that any information you provide can be matched at both timepoints, while remaining anonymous.

How long will my part in the study last?

You will be asked to participate in this study for 2 weeks. Following completion of the first questionnaire, participants will be contacted 2 weeks later to complete the follow-up questionnaire. The first questionnaire will take approximately 4 minutes and the follow-up questionnaire will take approximately 10 minutes to complete.

What are the possible benefits in taking part?

This study will introduce you to an app which you may find beneficial for taking your preventer inhaler and managing your asthma, and which you may continue to use after the study. You will have contributed to important research in your condition, which will be valuable for the wider community of young adults living with asthma.

What are the possible disadvantages and risks of taking part?

There are no anticipated disadvantages or risks to taking part in this study. As with all mobile apps, the maker of the commercial app will have a privacy policy concerning your data. We do not control this policy. We would encourage you to make yourself familiar with this before you agree to download the app. None of the individual level data that we collect will be shared with anyone outside of the University and we will not have access to any data recorded by the app.

What happens at the end of the study?

We would be happy to share the results of the study with you once it is complete if they are of interest to you. It should be noted that information collected in this research study may be submitted for publication in a scientific journal. This process could take up to two years. All information obtained from this study will be completely anonymous and will not identify you in any way.

What happens if I change my mind during the study?

You are entitled to change your mind about taking part in this study at any time. You may withdraw your participation at any point without disadvantage or penalty. You will not be required to provide a reason for withdrawal.

Who do I contact for more information or if I have further concerns?

If you would like more information or have any concerns, feel free to contact the researcher, XXX XXX, by email at XXXXXXX.

If you have any concerns about this study and wish to contact someone in confidence, you may contact: **XXX XXX.**

Your participation in this study is greatly appreciated. If after reading this information you decide to take part, please provide your consent in the following section.

Thank you.

Researcher: XXX XXX

Supervisors: XXX XXX, XXX XXX & XXX XXX
