# Supplementary material for: A Smartphone App to Support Adherence to Inhaled Corticosteroids in Young Adults With Asthma: Multi-Methods Feasibility Study
Source: JMIR Form Res. 2021 Sep 1;5(9):e28784. doi: 10.2196/28784 (PMC8444040; doi:10.2196/28784)
Supplement: Multimedia Appendix 2 [file formative_v5i9e28784_app2.pdf]

Study consent form

If you wish to take part in this study, please provide your consent below by ticking the appropriate boxes. You are free to withdraw at any time and without giving a reason.

I confirm that I am 18-30 years of age *

- Yes

I confirm that I have received an asthma diagnosis from a doctor or other healthcare professional *

- Yes

I confirm that I am currently prescribed inhaled corticosteroids, i.e. a preventer inhaler *

- Yes

I am satisfied that I understand the information provided and have had enough time to consider the information *

- Yes

I confirm that I have read the information sheet for the above study and have had the opportunity to ask questions *

- Yes

I understand that my participation is voluntary and that I am free to withdraw at any time, without providing a reason and without consequence *

- Yes

I consent to take part in this study *

- Yes
