# Supplementary material for: A Smartphone App to Support Adherence to Inhaled Corticosteroids in Young Adults With Asthma: Multi-Methods Feasibility Study
Source: JMIR Form Res. 2021 Sep 1;5(9):e28784. doi: 10.2196/28784 (PMC8444040; doi:10.2196/28784)
Supplement: Multimedia Appendix 6 [file formative_v5i9e28784_app6.pdf]

## Checklist for Reporting Results of Internet E-surveys (CHERRIES)

| ***Item category*** | ***Checklist item*** | ***Explanation*** | |
|---|---|---|---|
| **Design** | | | |
| | Describe survey design | Describe target population, sample frame. Is the sample a convenience sample? (In “open” surveys this is most likely.) | We aimed to collect data from young adults (18-30 years) living with asthma and currently prescribed a form of ICS, from the Republic of Ireland. An open survey was administered at baseline and 2-week follow-up, via LimeSurvey, an online platform (pg. 6). |
| **IRB (Institutional Review Board) approval and informed consent process** | | | |
| | IRB approval | Mention whether the study has been approved by an IRB. | Ethical approval was granted by the relevant University Ethics Committee (reference number: 20-Jan-13) on February 18, 2020 (pg. 2) |
| | Informed consent | Describe the informed consent process. Where were the participants told the length of time of the survey, which data were stored and where and for how long, who the investigator was, and the purpose of the study? | This information was provided in the participant study information sheet (Appendix 1). Informed consent was obtained electronically (pg. 2). |
| | Data protection | If any personal information was collected or stored, describe what mechanisms were used to protect unauthorised access. | Data were collected and stored in line with University guidance and GDPR, e.g. password-protected computers. Participant email addresses were collected in a separate form so this could not be linked to any other data they provided in the study (Appendix 1). |
| **Development and testing** | | | |
| | Development and testing | State how the survey was developed, including whether the usability and technical functionality of the electronic had been tested before fielding the questionnaire. | The surveys were designed as open surveys using LimeSurvey. Firstly, the research team sought and modified relevant questionnaires from similar studies and psychometric scales from the literature, to compose the baseline and follow-up questionnaires. Secondly, PPI contributors were asked to provide feedback on the proposed surveys (pg. 3). |

| | | | |
|---|---|---|---|
| | Open survey versus closed survey | An open survey is a survey open for each visitor of a site, while a closed survey is only open to a sample which the investigator knows (password-protected survey). | This survey was open to all participants. |
| | Contact mode | Indicate whether or not the initial contact with the potential participants was made on the Internet. (Investigators may also send out questionnaires by mail and allow for Web-based data entry). | Participants were recruited through social media, University circular mail where a link to the survey was provided. Additionally, participants were recruited through General Practice (GP) sites. The usernames/handles of the study's social medica accounts were provided in GP letters, where prospective participants could visit and access a link to the survey. A link to the follow-up survey was sent to participants' email addresses (pg. 6). |
| | Advertising the study | How/where was the study announced or advertised? Some examples are offline media (newspapers), or online (mailing lists – If yes, which ones?) or banner ads (Where were these ads posted and what did they look like?). It is important to know the wording of the announcement as it will heavily influence who chooses to participate. | Recruitment and advertisement strategies are outlined on pg. 4. |
| **Survey administration** | | | |
| | Web/E-mail | State the type of e-survey (e.g. one posted on a Web site, or one sent out through email). If it is an e-mail survey, were the responses entered manually into a database, or was there an automatic method for capturing responses? | Both the baseline and follow-up surveys were web-based. A link to the follow-up survey was sent to participants' email addresses (pg. 6). |
| | Context | Describe the Web site (for mailing list/newsgroup) in which the survey was posted. What is the Web site about, who is visiting it, what are visitors normally looking for? Discuss to what degree the content of the Web site could pre-select the sample or influence the results. | Recruitment and advertisement strategies are outlined on pg. 4. A link to the baseline survey was included in all advertisements and a link to the follow-up survey was included in emails sent from the researcher to participants. The survey was available on LimeSurvey, an open source |

| | | | |
|---|---|---|---|
| | | For example, a survey about vaccination on an anti-immunization Web site will still have different results from a Web survey conducted on a government Web site. | online survey software: https://www.limesurvey.org/ |
| | Mandatory/voluntary | Was it a mandatory survey to be filled in by every visitor who wanted to enter the Web site, or was it a voluntary survey? | Participation in the baseline and follow-up surveys were completely voluntary. Participants had the right to leave the survey at any time (Appendix 1). |
| | Incentives | Were any incentives offered (e.g. monetary, prizes, or non-monetary incentives such as an offer to provide the survey results?). | Participants were not compensated or offered any incentive to take part in the study. As an incentive to support recruitment, GPs were offered an Irish Medical Council-eligible audit template of clinically relevant Global Initiative for Asthma (GINA) guidelines that was conducive to fulfilling their annual audit requirements (pg. 3-4). |
| | Time/Date | In what timeframe were the data collected? | All data were collected from September to December 2020 (pg. 2) |
| | Randomization of items or questionnaires | To prevent biases items can be randomized or alternated. | Randomization of items/questionnaires was not applied. |
| | Adaptive questioning | Use adaptive questioning (certain items, or only conditionally displayed based on responses to other items) to reduce number and complexity of the questions. | Adaptive questioning was not applied. |
| | Number of Items | What was the number of questionnaire items per page? The number of items is an important factor for the completion rate. | The number of questions averaged between 5-6 per page. |
| | Number of screens (pages) | Over how many pages was the questionnaire distributed? The number of items is an important factor for the completion rate. | The baseline and follow-up surveys were 9 and 19 pages, respectively. |
| | Completeness check | It is technically possible to do consistency or completeness checks before the questionnaire is submitted. Was this done, and if "yes", how (usually JAVAScript)? An alternative is to | Questions were not mandatory. Participants were asked to complete all questions, however f they did not wish to answer a question(s) in any section, they could leave it blank. Consistency |

| | | | |
|---|---|---|---|
| | | check for completeness after the questionnaire has been submitted (and highlight mandatory items). If this has been done, it should be reported. All items should provide a non-response option such as "not applicable" or "rather not say", and selection of one response option should be enforced. | and completeness checks were not performed. |
| | Review step | State whether respondents were able to review and change their answers (e.g. through a Back button or a Review step which displays a summary of the responses and asks the respondents if they are correct). | Participants had the option to go back and review/change answers. |
| **Response rates** | | | |
| | Unique site visitor | If you provide view rates or participation rates, you need to define how you determined a unique visitor. There are different techniques available, based on IP addresses or cookies or both. | Unique site visitors were not determined. |
| | View rate (Ratio of unique survey visitors/unique site visitors) | Requires counting unique visitors to the first page of the survey, divided by the number of unique site visitors (not page views!). It is not unusual to have view rates of less than 0.1 % if the survey is voluntary. | Not applicable. |
| | Participation rate (Ratio of unique visitors who agreed to participate/unique first survey page visitors) | Count the unique number of people who filled in the first survey page (or agreed to participate, for example by checking a checkbox), divided by visitors who visit the first page of the survey (or the informed consents page, if present). This can also be called "recruitment" rate. | A total of 122 participants provided informed consent and completed the baseline survey (pg. 8). |
| | Completion rate (Ratio of users who finished the survey/users who agreed to participate) | The number of people submitting the last questionnaire page, divided by the number of people who agreed to participate (or submitted the first survey page). This is only relevant if there is a separate "informed consent" | A total of 59/122 (48.4%) participants completed the follow-up survey (pg. 8). |

| | | | |
|---|---|---|---|
| | | page or if the survey goes over several pages. This is a measure for attrition. Note that “completion” can involve leaving questionnaire items blank. This is not a measure for how completely questionnaires were filled in. (If you need a measure for this, use the word “completeness rate”.) | |
| **Preventing multiple entries from the same individual** | | | |
| | Cookies used | Indicate whether cookies were used to assign a unique user identifier to each client computer. If so, mention the page on which the cookie was set and read, and how long the cookie was valid. Were duplicate entries avoided by preventing users access to the survey twice; or were duplicate database entries having the same user ID eliminated before analysis? In the latter case, which entries were kept for analysis (eg, the first entry or the most recent)? | Participants were asked to create their own unique personal identifying code so that any information they provided could be matched at both timepoints, while remaining anonymous (Appendix 1). |
| | IP check | Indicate whether the IP address of the client computer was used to identify potential duplicate entries from the same user. If so, mention the period of time for which no two entries from the same IP address were allowed (eg, 24 hours). Were duplicate entries avoided by preventing users with the same IP address access to the survey twice; or were duplicate database entries having the same IP address within a given period of time eliminated before analysis? If the latter, which entries were kept for analysis (eg, the first entry or the most recent)? | The research team checked for and removed any duplicates, by checking for multiple entries from the same personal identifying code. |
| | Log file analysis | Indicate whether other techniques to analyze the log file for identification of multiple entries were used. If so, please describe. | Not applicable. |
| | Registration | In “closed” (non-open) surveys, users need to login first and it is | Not applicable. |

| | | | |
|---|---|---|---|
| | | easier to prevent duplicate entries from the same user. Describe how this was done. For example, was the survey never displayed a second time once the user had filled it in, or was the username stored together with the survey results and later eliminated? If the latter, which entries were kept for analysis (eg, the first entry or the most recent)? | |
| **Analysis** | | | |
| | Handling of incomplete questionnaires | Were only completed questionnaires analyzed? Were questionnaires which terminated early (where, for example, users did not go through all questionnaire pages) also analyzed? | Data were analyzed for all questionnaires, i.e. those completed and not completed. |
| | Questionnaires submitted with an atypical timestamp | Some investigators may measure the time people needed to fill in a questionnaire and exclude questionnaires that were submitted too soon. Specify the timeframe that was used as a cut-off point, and describe how this point was determined. | Timestamps were recorded however these were not used as a cut-off point to exclude responses. |
| | Statistical correction | Indicate whether any methods such as weighting of items or propensity scores have been used to adjust for the non-representative sample; if so, please describe the methods. | We did not use any statistical correction methods to adjust for a potentially non-representative sample. |
