# Supplementary material for: A Smartphone App to Support Adherence to Inhaled Corticosteroids in Young Adults With Asthma: Multi-Methods Feasibility Study
Source: JMIR Form Res. 2021 Sep 1;5(9):e28784. doi: 10.2196/28784 (PMC8444040; doi:10.2196/28784)
Supplement: Multimedia Appendix 7 [file formative_v5i9e28784_app7.pdf]

Rationale for Go- and No-Go study progression criteria and each threshold

In line with relevant guidance [1] the research team identified the key uncertainties that needed to be assessed in relation to the feasibility of using AsthmaMD and examining its efficacy in a future trial in young adults. These key uncertainties were identified from the relevant literature and combined research team expertise in young adult recruitment and retention, digital intervention development and evaluation and relevant parameters of feasibility studies. Following an iterative process of discussion and revision, the research team agreed the below Go- and No-Go progression criteria for the study and how these criteria would be measured and interpreted accordingly.

We acknowledge that there may be alternative appropriate measures and thresholds for these criteria. However, based on our context-specific combined expertise we propose the following potential approach to interpret the outcomes of this study in determining the feasibility of recruiting and retaining young adults to a future trial and the feasibility of the AsthmaMD app.

1. Feasibility of participant recruitment

Can ≥ 74 participants be recruited to the study?

The minimum number of participants required to complete this study was 59. The anticipated rate of attrition for this study was 25%. This was based on attrition rates in similar app feasibility studies with a 2-week follow-up which ranged from 4-25% [2-4]. The research team decided to use the upper estimate of 25% as opposed to the average of these rates due to the challenges involved in recruiting and retaining young adults. Additionally, this is based on a recent similar study of an asthma self-management app in young people which reported 25% attrition [5]. To allow for 25% attrition, 74 participants was the minimum target sample size for this study and therefore was selected as the 'Green: Proceed' threshold.

To determine the 'Amber: Amend' and 'Red: Stop' thresholds for this criterion, the research team used the average and lowest rate of 10 and 4% attrition, respectively, from the aforementioned similar studies.

2. Feasibility of participant retainment

Can ≥ 59 (75%) participants be retained in the study until completion?

At least 59 participants were needed to complete the study. This was based on published recommendations, which state that if a problem exists with a 5% probability in a study participant it would be identified in a sample of 59 participants [6]. Accordingly, 59 participants were selected as the 'Green: Proceed' threshold. To inform the other thresholds for this criterion, the research team examined the retained, final sample size in app feasibility studies using similar methods with a 2-week follow-up [3, 4, 7]. The mean final sample size from these studies was 40 participants. Based on this and the study's target sample size, the research team used 40 participants as the 'Red: Stop' threshold. The team selected 50 participants as the 'Amber: Amend' threshold representing an approximate midpoint between these sample sizes.

3. Usability of 'AsthmaMD'

Will the app receive a mean SUS score >68?

The System Usability Scale [SUS; 8] was used to measure the perceived usability of the AsthmaMD app at follow-up. An SUS score greater than 68 is considered above average [9, 10] and so informed our threshold for 'Green: Proceed'. SUS scores have also been translated into letter grades to enable

effective communication of its results to a range of stakeholders [10]. This grading scale was used to determine the remaining usability thresholds. An SUS score of 0-52 was assigned an F grade, and therefore was selected as the threshold for 'Red: Stop'. A score of 63-65 was assigned a C- grade and thus was selected as the threshold for 'Amber: Amend'. Additionally, qualitative data relating to the acceptability of AsthmaMD will be considered.

4. Acceptability of 'AsthmaMD'

Will the app receive a mean score ≥ 5 for overall user satisfaction?

or

Will ≥ 30% of participants say yes to 3/5 acceptability-related questions?

Acceptability of AsthmaMD was assessed at follow-up by two measures adapted from a recent feasibility study of an app for medication adherence in a chronic condition [11]. Firstly, acceptability was assessed from participants ratings of their overall user satisfaction with the app on a scale of 1-10. The research team selected ≥5/10 as the threshold for 'Green: Proceed', as they logically agreed that this midpoint of the scale would indicate the sufficient user satisfaction with the app to proceed with its evaluation. Following this reasoning in a consecutively descending order, the team selected ≥4/10 and <4/10 as the 'Amber: Amend' and 'Red: Stop' thresholds, respectively.

Secondly, acceptability was measured by participant responses to whether the app: (1) made you more aware of your adherence, (2) made you more adherent, (3) made you more confident in managing your ICS, (4) reduces the stress in managing your ICS, and (5) if app notifications were not annoying. The research team used the following as the 'Green: Proceed' threshold: ≥30% of participants agreeing to 3/5 of these questions. This was informed from a recent mHealth feasibility and acceptability study which used a threshold of 30% of participants responding positively to similar measures to define acceptability of the intervention [12]. The team logically agreed that this percentage of participants agreeing to at least the majority of these questions, i.e., 3/5 questions, would be sufficient. Again, following a consecutive descending order the team selected ≥30% agreeing to 2/5 questions and <30% agreeing to <2/5 questions as the 'Amber: Amend' and 'Red: Stop' thresholds, respectively. Finally, relevant qualitative data will be taken into consideration.

5. Feasibility of 'AsthmaMD'

Did ≥ 30% of participants use the app ≥ 1 day per week?

or

Would ≥ 30% of participants continue to use the app after the study?

Feasibility of AsthmaMD was assessed at follow-up by the following two commonly used measures in similar studies of mHealth apps [11, 12]. Firstly, feasibility was measured by participants frequency of app use in days per week throughout the study period. The research team selected ≥30% of participants using the app ≥1 day per week as the threshold for 'Green: Proceed'. As previously outlined, this 30% threshold of participants has been used to determine the feasibility of mHealth apps from similar measures [12]. We acknowledge that using the app ≥1 day per week may be considered relatively infrequent use in certain chronic conditions. However, given the symptomatic/asymptomatic nature of asthma and its variability over time due to a range of factors such as adherence, physical activity, allergen or irritant exposure, seasonal changes or viral respiratory infections [13], the team agreed that this is an appropriate threshold for an asthma app, as frequency of use will likely vary depending on users' needs and preferences at different times. Similarly, the

‘Amber: Amend’ and ‘Red: Stop’ thresholds were based on a logically descending percentage of participants; ≥25% and <25% used the app 1 day per week, respectively.

Secondly, feasibility was measured by whether participants intended to continue using the app after the study. The team also selected ≥30% participants indicating that they would continue to use the app as the ‘Green: Proceed’ threshold. This was based on the 30% threshold previously employed to define feasibility in similar studies [12]. Once again, the team followed a consecutively descending order to select the ‘Amber: Amend’ and ‘Red: Stop’ thresholds for this measure; ≥25% and <25% indicating they would continue to use the app, respectively. Feasibility-related data will also be taken into account.

References

1. Eldridge, S.M., et al., *Defining feasibility and pilot studies in preparation for randomised controlled trials: development of a conceptual framework.* PloS one, 2016. **11**(3): p. e0150205.
2. Degroote, L., et al., *Acceptability and feasibility of the mHealth intervention 'MyDayPlan'to increase physical activity in a general adult population.* BMC public health, 2020. **20**(1): p. 1-12.
3. Przybyla, S.M., et al., *Feasibility and acceptability of a smartphone app for daily reports of substance use and antiretroviral therapy adherence among HIV-infected adults.* AIDS research and treatment, 2016. **2016**.
4. Woods, L.S., et al., *Patients' Experiences of Using a Consumer mHealth App for Self-Management of Heart Failure: Mixed-Methods Study.* JMIR human factors, 2019. **6**(2): p. e13009.
5. Davis, S.R., et al., *A consumer designed smartphone app for young people with asthma: pilot of engagement and acceptability.* Journal of Asthma, 2019: p. 1-9.
6. Viechtbauer, W., et al., *A simple formula for the calculation of sample size in pilot studies.* Journal of clinical epidemiology, 2015. **68**(11): p. 1375-1379.
7. Appleton, K.M., et al., *An Interactive Mobile Phone App (SMART 5-A-DAY) for Increasing Knowledge of and Adherence to Fruit and Vegetable Recommendations: Development and Pilot Randomized Controlled Trial.* JMIR mHealth and uHealth, 2019. **7**(11): p. e14380.
8. Brooke, J., *SUS: a "quick and dirty'usability.* Usability evaluation in industry, 1996: p. 189.
9. Sauro, J., *A practical guide to the system usability scale: Background, benchmarks & best practices*. 2011: Measuring Usability LLC.
10. Sauro, J. and J.R. Lewis, *Quantifying the user experience: Practical statistics for user research*. 2016: Morgan Kaufmann.
11. Huang, Z., et al., *A smartphone app to improve medication adherence in patients with type 2 diabetes in Asia: feasibility randomized controlled trial.* JMIR mHealth and uHealth, 2019. **7**(9): p. e14914.
12. Zhang, M., et al., *A Smartphone Attention Bias App for Individuals With Addictive Disorders: Feasibility and Acceptability Study.* JMIR mHealth and uHealth, 2019. **7**(9): p. e15465.
13. Asthma, G.I.f., *Global Strategy for Asthma Management and Prevention*. 2020.
