# Supplementary material for: A Smartphone App to Support Adherence to Inhaled Corticosteroids in Young Adults With Asthma: Multi-Methods Feasibility Study
Source: JMIR Form Res. 2021 Sep 1;5(9):e28784. doi: 10.2196/28784 (PMC8444040; doi:10.2196/28784)
Supplement: Multimedia Appendix 8 [file formative_v5i9e28784_app8.pdf]

Table S1. Go- and No-Go progression criteria

	**Go – Proceed with RCT**	**Amend – Proceed with changes**	**Stop – Do not proceed unless changes are possible**
**1.Feasibility of participant recruitment**<br>Can ≥74 participants be recruited to take part in the study?	≥74 participants	≥65 participants	<61 participants
**2. Feasibility of participant retention**<br>Can ≥59 participants be retained in the study until completion?	≥59 retained	≥50 retained	<40 retained
**3. Usability of 'AsthmaMD'**<br>Will the app receive a mean SUS score >68?<br>*and*<br>Interpretation of qualitative data relating to usability	SUS >68<br>*and*<br>App judged highly usable	SUS ≥63<br>*and*<br>App judged usable	SUS <52<br>*and*<br>App judged possibly usable
**4. Acceptability of 'AsthmaMD'**<br>Will the app receive a mean score ≥5 for overall user satisfaction?<br>*or*<br>Will ≥30% of participants agree to 3/5 acceptability-related questions?<br>*and*<br>Interpretation of qualitative data relating to acceptability	≥5 overall satisfaction<br>*or*<br>≥30% agree to 3/5 questions<br>*and*<br>App judged highly acceptable	≥4 overall satisfaction<br>*or*<br>≥30% agree to 2/5 questions<br>*and*<br>App judged acceptable	<4 overall satisfaction<br>*or*<br><30% agree to <2/5 questions<br>*and*<br>App judged possibly acceptable
**5. Feasibility of 'AsthmaMD'**<br>Did ≥30% of participants use the app ≥1 day per week?<br>*or*<br>Would ≥30% of participants continue to use the app after the study?<br>*And*<br>Interpretation of qualitative data relating to feasibility	≥30% used app ≥1 day/week<br>*or*<br>≥30% continue to use app<br>*And*<br>App judged highly feasible	≥25% used app ≥1 day/week<br>*or*<br>≥25% continue to use app<br>*and*<br>App judged feasible	<20% used app <1 day/week<br>*or*<br><20% continue to use app<br>*and*<br>App judged possibly feasible
