# Supplementary material for: A Smartphone App to Support Adherence to Inhaled Corticosteroids in Young Adults With Asthma: Multi-Methods Feasibility Study
Source: JMIR Form Res. 2021 Sep 1;5(9):e28784. doi: 10.2196/28784 (PMC8444040; doi:10.2196/28784)

Baseline questionnaire

Demographics

This section will ask about your demographics, such as gender and age.

1. Please state your age (in years)

Please write your answer here: _______________

2. Please select your gender

- Female
- Male
- Prefer not to say
- Other:

3. Please indicate your highest level of education

- Second level
- Third level
- Postgraduate

4. Please indicate your ethnicity

- White: Irish
- White: Irish Traveller
- Any other white background
- Black/Black Irish
- Asian/Asian Irish
- Mixed
- Other:

5. Do you currently hold a medical card?

- Yes
- No

6. Do you currently hold a GP visit card?

- Yes
- No

7. Where did you hear about this study?

Please choose **all** that apply:

- Facebook
- Twitter
- General Practitioner (GP) / Nurse
- University student mailer
- Advertisements on University campus

- Advertisements in Galway region
- Sports club
- Word of mouth
- Other:

Asthma characteristics

This section will ask about your asthma characteristics such as the number of years since your asthma diagnosis, the name and prescribed dose of your preventer inhaler, and any asthma exacerbations in the past year.

1. Please indicate the number of years since your asthma diagnosis

- <1 year
- 1-2 years
- 2-5 years
- 5-10 years
- 10+ years

2. What is the name of your preventer inhaler?

- Becotide
- Pulmicort
- Flixotide
- Don't know
- Other:

3. How many times per day are you prescribed to take your preventer inhaler?

- Once a day
- Twice a day
- Don't know

4. Have you visited the hospital emergency department because of an asthma exacerbation in the past year?

- Yes
- No

5. Have you been admitted to hospital because of an asthma exacerbation in the past year?

- Yes
- No

6. Have you been treated with oral steroids for your asthma in the past year? (Oral steroids are tablets prescribed by your doctor for a brief time, e.g. 5 days, if you have serious worsening of asthma symptoms. Examples include: Prednisone, Medrol, Prelone.)

- Yes
- No

Questions about using your preventer inhaler

Most people find a way of using their medicines which suits them. We are interested in what method suits you. Here are some ways in which people have said that they use their preventer treatment. For each statement, please tick the answer that best applies to you.

© R Horne University of Brighton 1999

1. I only use it when I need it

	Always	Often	Sometimes	Rarely	Never

2. I only use it when I feel breathless

	Always	Often	Sometimes	Rarely	Never

3. I decide to miss out a dose

	Always	Often	Sometimes	Rarely	Never

4. I try to avoid using it

	Always	Often	Sometimes	Rarely	Never

5. I forget to take it

	Always	Often	Sometimes	Rarely	Never

6. I alter the dose

	Always	Often	Sometimes	Rarely	Never

7. I stop taking it for a while

	Always	Often	Sometimes	Rarely	Never

8. I use it as a reserve, if my other treatment doesn't work.

	Always	Often	Sometimes	Rarely	Never

9. I use it before doing something which might make me breathless

	Always	Often	Sometimes	Rarely	Never

10. I take less than instructed

	Always	Often	Sometimes	Rarely	Never

Asthma Control Test™ ©. Copyright, QualityMetric Incorporated 2002, 2004. All rights reserved. Asthma Control Test™ © is a trademark of QualityMetric Incorporated. United Kingdom (English) version.

Asthma Control Test™

This survey was designed to help you describe your asthma and how your asthma affects how you feel and what you are able to do. To complete it, please select the box that best describes your answer.

1. During the <u>last 4 weeks</u>, how much of the time has your <u>asthma</u> kept you from getting as much done at work, school or home?

	All of the time	Most of the time	Some of the time	A little of the time	None of the time

2. During the <u>last 4 weeks</u>, how often have you had shortness of breath?

	More than once a day	Once a day	3 to 6 times a week	Once or twice a week	Not at all

3. During the <u>last 4 weeks</u>, how often have your <u>asthma</u> symptoms (wheezing, coughing, shortness of breath, chest tightness or pain) woken you up at night or earlier than usual in the morning?

	4 or more nights a week	2 to 3 nights a week	Once a week	Once or Twice	Not at all

4. During the last 4 weeks, how often have you used your rescue inhaler or nebuliser medication (such as Salbutamol)?

	3 or more times per day	Once or twice per day	2 or 3 times per week	Once a week or less	Not at all

5. How would you rate your asthma control during the last 4 weeks?

	Not Controlled at all	Poorly Controlled	Somewhat Controlled	Well Controlled	Completely Controlled

Smartphone and app experience

This section will ask about your experience and use of smartphones and apps.

1. Please indicate the number of years that you have been using a smartphone

- <1 year
- 1-3 years
- 3-5 years
- 5+ years
- 10+ years

2. How many hours per day do you use smartphone apps?

- <1 hour
- 1-3 hours
- 3-5 hours
- 5+ hours

3. Have you ever used a health app(s) before? (e.g. for physical activity, healthy diet, quitting smoking)

- Yes
- No

4. If so, what was this app for?

- Physical activity
- Healthy diet
- Quitting smoking
- Taking medication
- Other:

5. Have you ever used an asthma app(s) before this study?

- Yes
- No

6. If so, please state the name of this asthma app(s)

Please write your answer here: ______________________

7. Where did you hear about this app(s)?

Please choose **all** that apply:

- Peers
- Family
- Social media
- Healthcare professional
- Other:

8. If so, what did you use this asthma app(s) for?

Please choose **all** that apply:

- To take your inhalers
- To record/monitor your symptoms
- To record/monitor your triggers
- To complete an asthma action plan
- Other:

9. Why did you decide to use this app?

Please write your answer here: _____________________

10. Are you currently using an asthma-related app(s)?

- Yes
- No

11. Were you aware of any asthma app(s) before this study?

- Yes
- No

12. If so, what was the name of this app(s)?

Please write your answer here: _____________________

13. Where did you hear about this app(s)?

Please choose **all** that apply:

- Peers
- Family
- Social media
- Healthcare professional

- Other:

Thank you for completing this questionnaire.

- Please provide your email address here. This is so we can contact you to complete the follow-up questionnaire.
- Please download the 'AsthmaMD' app today and use it as you wish for the next 2 weeks (see screenshot of logo and homescreen of app below).
- In 2 weeks, we will email you to complete a follow-up questionnaire asking about your experience of using the app.

Please note that the app may not alert you if your phone is switched off or out of battery.

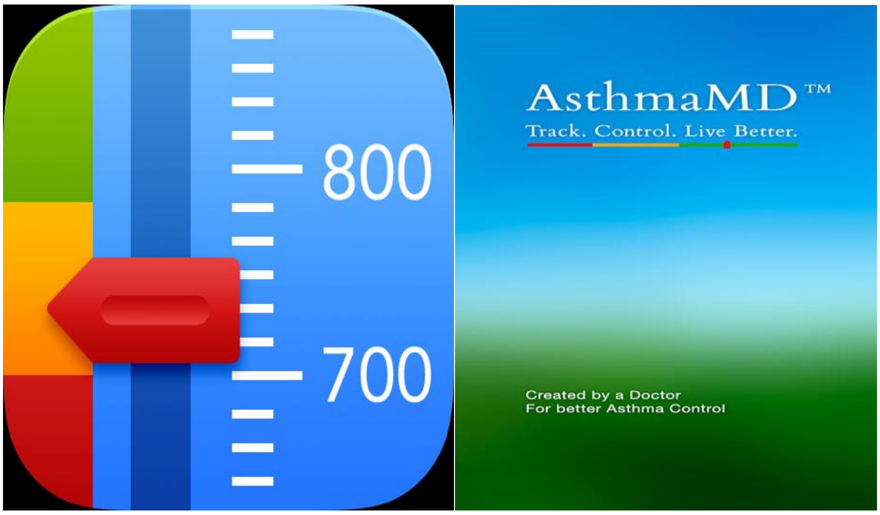

Supplement: Multimedia Appendix 9 [file formative_v5i9e28784_app9.pdf]
