# Supplementary material for: A Smartphone App to Support Adherence to Inhaled Corticosteroids in Young Adults With Asthma: Multi-Methods Feasibility Study
Source: JMIR Form Res. 2021 Sep 1;5(9):e28784. doi: 10.2196/28784 (PMC8444040; doi:10.2196/28784)
Supplement: Multimedia Appendix 10 [file formative_v5i9e28784_app10.pdf]

Follow-up Questionnaire

Thank you for completing the first questionnaire in this study. On completing the first questionnaire you were asked to download and use the 'AsthmaMD' app for 2 weeks. This follow-up questionnaire will ask about your experience of using the app and will take approximately 10 minutes to complete.

Your participation in this follow-up questionnaire is also completely optional and you are free to withdraw at any time.

Thank you for your time.

Asthma Control Test™ ©. Copyright, QualityMetric Incorporated 2002, 2004. All rights reserved. Asthma Control Test™ © is a trademark of QualityMetric Incorporated. United Kingdom (English) version.

Asthma Control Test™

This survey was designed to help you describe your asthma and how your asthma affects how you feel and what you are able to do. To complete it, please select the box that best describes your answer.

1. During the last 4 weeks, how much of the time has your asthma kept you from getting as much done at work, school or home?

	All of the time	Most of the time	Some of the time	A little of the time	None of the time

2**. During the last 4 weeks, how often have you had shortness of breath?**

	More than once a day	Once a day	3 to 6 times a week	Once or twice a week	Not at all

3. During the last 4 weeks, how often have your asthma symptoms (wheezing, coughing, shortness of breath, chest tightness or pain) woken you up at night or earlier than usual in the morning?

	4 or more nights a week	2 to 3 nights a week	Once a week	Once or Twice	Not at all

4. During the last 4 weeks, how often have you used your rescue inhaler or nebuliser medication (such as Salbutamol)?

	3 or more times per day	Once or twice per day	2 or 3 times per week	Once a week or less	Not at all

5. How would you rate your asthma control during the last 4 weeks?

	Not Controlled at all	Poorly Controlled	Somewhat Controlled	Well Controlled	Completely Controlled

Questions about using your preventer inhaler

Most people find a way of using their medicines which suits them. We are interested in what method suits you. Here are some ways in which people have said that they use their preventer treatment. For each statement, please tick the answer that best applies to you.

© R Horne University of Brighton 1999

1. I only use my preventer inhaler when I need it

	Always	Often	Sometimes	Rarely	Never

2. I only use it when I feel breathless

	Always	Often	Sometimes	Rarely	Never

3. I decide to miss out a dose

	Always	Often	Sometimes	Rarely	Never

4. I try to avoid using it

	Always	Often	Sometimes	Rarely	Never

5. I forget to take it

	Always	Often	Sometimes	Rarely	Never

6. I alter the dose

	Always	Often	Sometimes	Rarely	Never

7. I stop taking it for a while

	Always	Often	Sometimes	Rarely	Never

8. I use it as a reserve, if my other treatment doesn't work

	Always	Often	Sometimes	Rarely	Never

9. I use it before doing something which might make me breathless

	Always	Often	Sometimes	Rarely	Never

10. I take it less than instructed

	Always	Often	Sometimes	Rarely	Never

App Usability Scale

This section will ask about how easy you found the app to use. Please rate your response from '1: Strongly Disagree' to '5: Strongly Agree'.

1. I think that I would like to use this app frequently.

	1: Strongly Disagree	2	3	4	5: Strongly Agree

2. I found the app unnecessarily complex.

	1: Strongly Disagree	2	3	4	5: Stongly Agree

3. I thought the app was easy to use.

	1: Strongly Disagree	2	3	4	5: Strongly Agree

4. I think that I would need the support of a technical person to be able to use this app.

	1: Strongly Disagree	2	3	4	5: Strongly Agree

5. I found the various functions in this app were well integrated.

	1: Strongly Disagree	2	3	4	5: Strongly Agree

6. I thought there was too much inconsistency in this app.

	1: Strongly Disagree	2	3	4	5: Strongly Agree

7. I would imagine that most people would learn this app very quickly.

	1: Strongly Disagree	2	3	4	5: Strongly Agree

8. I found the app very cumbersome (i.e. slow or complicated).

	1: Strongly Disagree	2	3	4	5: Strongly Agree

9. I felt very confident using the app.

	1: Strongly Disagree	2	3	4	5: Strongly Agree

10. I needed to learn a lot before I could get going with this app.

	1: Strongly Disagree	2	3	4	5: Strongly Agree

Use of individual app features

This section will ask you about which specific features you used in the app and which of these features you found useful.

1. Which of the following features did you use?

Please choose **all** that apply:

- **Enter Data:**
  - Peak Flow
  - FEV-1
  - Oximetry
  - Symptoms
  - Triggers
  - Medications: Edit action plan
  - Notes
- **Diary-chart:**
  - 1 week
  - 1 month
  - 3 months
  - All
  - Send Report

- **Reminders**
- **Action Plan**

- **Settings**:
- Account:
  - Profile
  - Sharing
- **Advanced settings:**
  - Enable notes
  - Enable FEV-1
  - Enable pulse oximeter
  - Change PEF zone
  - Change FEV1 zone
- **Other:**
  - AsthmaMD FAQs
- Video Tutorials:
  - Using your peak flow meter
  - Understanding asthma
  - Asthma medication simplified
  - AsthmaMD app tutorial

- About AsthmaMD

- License Agreement

1. Which of the following features did you find useful?

Please choose **all** that apply:

- **Enter Data:**
    - Peak Flow
    - FEV-1
    - Oximetry
    - Symptoms
    - Triggers
    - Medications: Edit action plan
    - Notes
- **Diary-chart:**
    - 1 week
    - 1 month
    - 3 months
    - All
    - Send Report

- **Reminders**
- **Action Plan**

- **Settings**:
- Account:
    - Profile
    - Sharing
- **Advanced settings:**
    - Enable notes
    - Enable FEV-1
    - Enable pulse oximeter
    - Change PEF zone
    - Change FEV1 zone
- **Other:**
    - AsthmaMD FAQs
- Video Tutorials:
    - Using your peak flow meter
    - Understanding asthma
    - Asthma medication simplified
    - AsthmaMD app tutorial

- About AsthmaMD
- License Agreement

Feasibility

This section will ask you about how often you used the app over the past 2 weeks.

1. In the past 2 weeks, how many days per week did you use the app?

- 0

- ○ 1
- ○ 2
- ○ 3
- ○ 4
- ○ 5
- ○ 6
- ○ 7

2. In the past 2 weeks, how many minutes per day did you use the app?

- ○ <5
- ○ 5-10
- ○ 10-15
- ○ 15-20
- ○ 20-25
- ○ 25-30
- ○ 30+

Acceptability

This section will ask about how you perceived the app, your attitude towards using it and overall satisfaction with it.

1. The app made you more aware of your adherence to your preventer inhaler.

	**Strongly Disagree**	**Disagree**	**Neutral**	**Agree**	**Strongly Agree**

2. The app made you more adherent to your preventer inhaler.

	**Strongly Disagree**	**Disagree**	**Neutral**	**Agree**	**Strongly Agree**

3. The app made you more confident in managing your preventer inhaler.

	**Strongly Disagree**	**Disagree**	**Neutral**	**Agree**	**Strongly Agree**

4. The app reduces the stress in managing your preventer inhaler.

	**Strongly Disagree**	**Disagree**	**Neutral**	**Agree**	**Strongly Agree**

5. The app is easy to use.

	Strongly Disagree	Disagree	Neutral	Agree	Strongly Agree

6. The app annoys you when the notification goes off.

	Strongly Disagree	Disagree	Neutral	Agree	Strongly Agree

7. Would you recommend the app to another person with asthma?

- Yes
- Not sure
- No

8. Will you continue to use the app after today?

- Yes
- Not sure
- No

9. Would you pay for this app?

- Yes
- Not sure
- No

10. On a scale of 1-10, with 10 being very satisfied, how would you rate your experience in using an app for managing your preventer inhaler?

	1: Very Dissatisfied	2	3	4	5	6	7	8	9	10: Very Satisfied

Additional questions

The following questions will ask about what you like/dislike about the app, any difficulties you encountered and improvements you would like to suggest.

1. What did you like most about the app?

Please write your answer here:

2. What did you like the least about the app?

Please write your answer here:

3. Did you experience any problems or difficulties with the app? If so, what were they?

Please write your answer here:

4. How would you improve this app? Is there any feature/function missing that you think would be relevant?

Please write your answer here:

5. Are there any other comments you would like to make in relation to your experience of using the app?

Please write your answer here:

Thank you for participating in this study.
