# Supplementary material for: A Smartphone App to Support Adherence to Inhaled Corticosteroids in Young Adults With Asthma: Multi-Methods Feasibility Study
Source: JMIR Form Res. 2021 Sep 1;5(9):e28784. doi: 10.2196/28784 (PMC8444040; doi:10.2196/28784)
Supplement: Multimedia Appendix 11 [file formative_v5i9e28784_app11.pdf]

Change user | Jane | Save

Date	5 Mar 2021 at 13:48
Peak Flow	991
FEV-1	9.0
Oximetry	95
Symptoms	Chest tightness
Triggers	Dust Mites; Exercise
Medications	Ventolin; Symbicor...
Notes	

Enter Data | Diary-chart | Reminders | Action Plan | Settings

Enter data

Jane

Cancel

WELL

- No cough, wheeze, chest tightness, or shortness of breath during the day or night
- Can do usual activities

WORSE

- Cough
- Wheeze
- Chest tightness
- Shortness of breath
- Waking at night due to asthma
- Can do some, but not all,

Enter Data | Diary-chart | Reminders | Action Plan | Settings

Change user

Jane

Send Report

1 W | 1 M | 3 M | All

5 March 2021

Worse

13:48

Symptoms: Chest tightness

Peak Flow:
991

Oximetry:
95

FEV-1:
9.0

Symptoms:
Chest tightness

Triggers:
Dust Mites; Exercise

Medications:
Ventolin; Symbicort

3 March 2021

Well

08:35

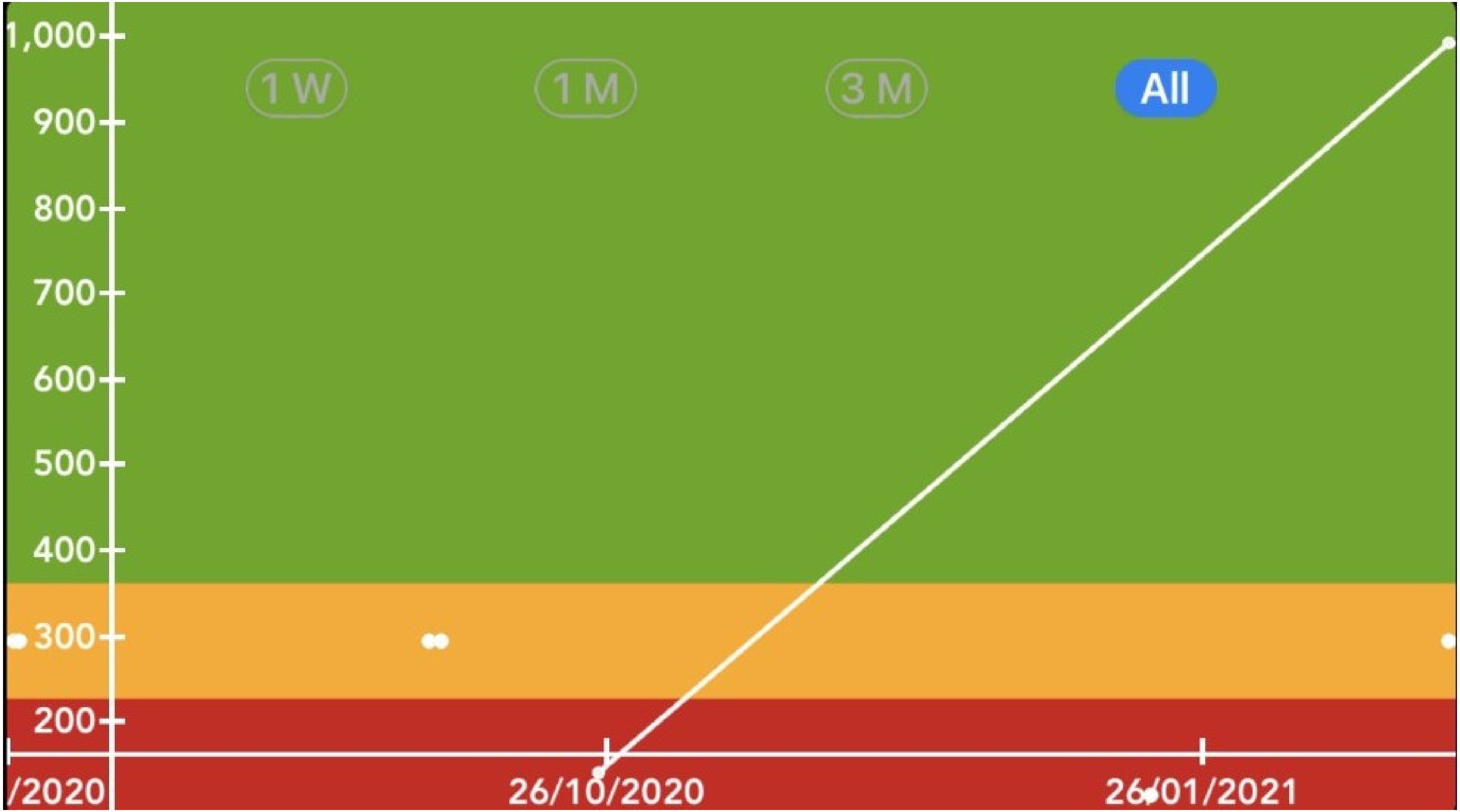
1,000
900
800
700
600
500
400
300
200
1 W
1 M
3 M
All
/2020
26/10/2020
26/01/2021

Change user

Jane

Ventolin 1 inh 1/day

Off Once a day **As prescribed**

First 8:00 >

Symbicort 2 puffs 2/days

Off **Once a day** As prescribed

First 8:00 >

Beclazone 2 puffs 2/days

Off Once a day As prescribed

Enter Data | Diary-chart | Reminders | Action Plan | Settings

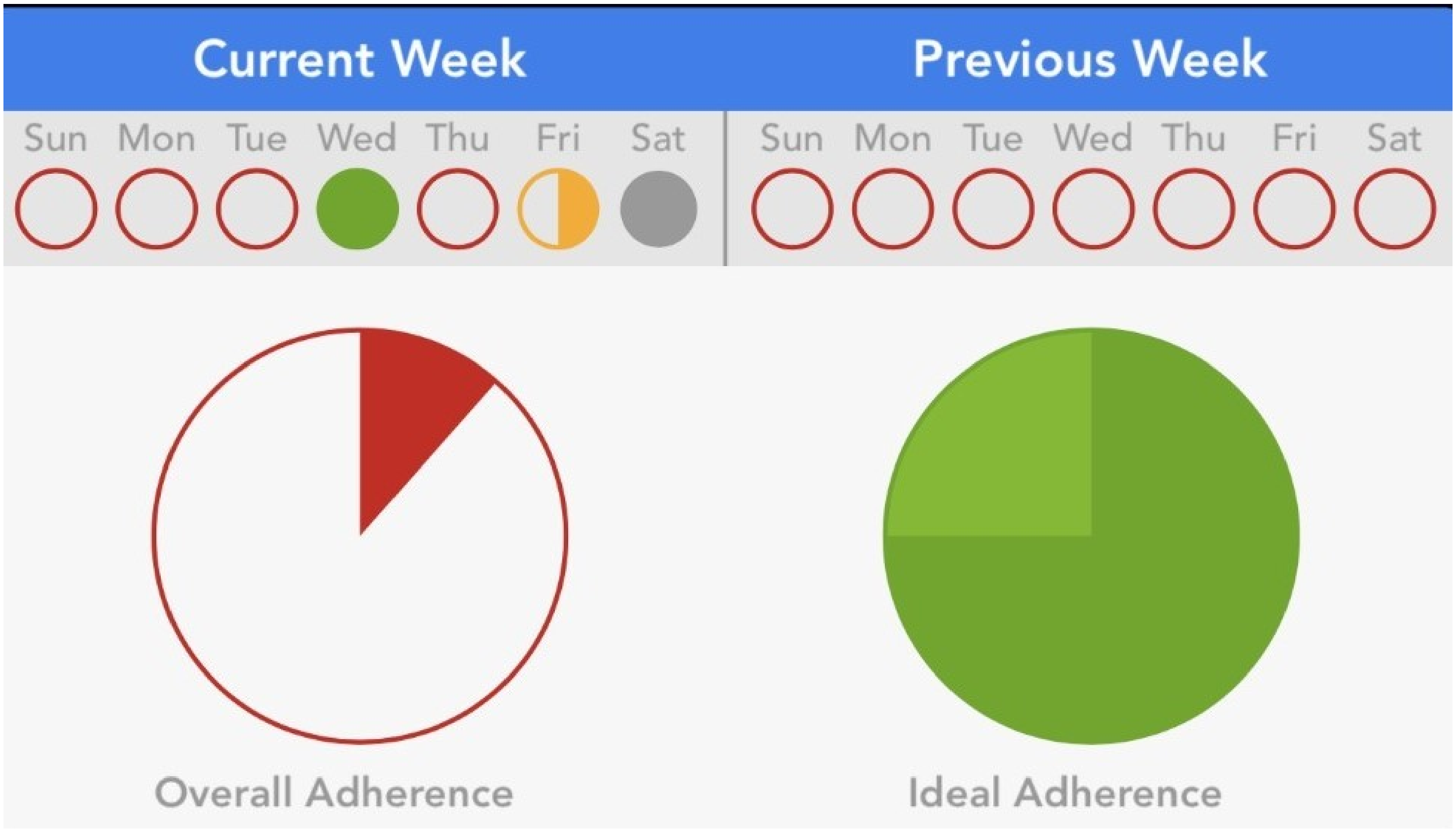
Current Week
Previous Week
Sun Mon Tue Wed Thu Fri Sat
Sun Mon Tue Wed Thu Fri Sat
Overall Adherence
Ideal Adherence

Caution! Continue taking every day controller medicines, and:

- Take 1 inh of **Ventolin** 1/day.
- Take 2 puffs of **Symbicort** 2/day, 2 puffs of **Beclazone** 2/day.
- Continue using quick relief medicine every 4 hours as needed.

DOING WELL

No cough, wheeze, chest tightness, or shortness of breath during the day or night.

Can do usual activities.

Peak Flow is 360 or more

every day:

ffs 2/days, **Beclazone** 2 puffs 2/days.

nh of **Ventolin** 1/day.

my asthma worse.
