# Supplementary figures and images for: A Smartphone App to Support Adherence to Inhaled Corticosteroids in Young Adults With Asthma: Multi-Methods Feasibility Study
Source: JMIR Form Res. 2021 Sep 1;5(9):e28784. doi: 10.2196/28784 (PMC8444040; doi:10.2196/28784)

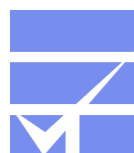

# CONSORT

TRANSPARENT REPORTING of TRIALS

## CONSORT 2010 Flow Diagram

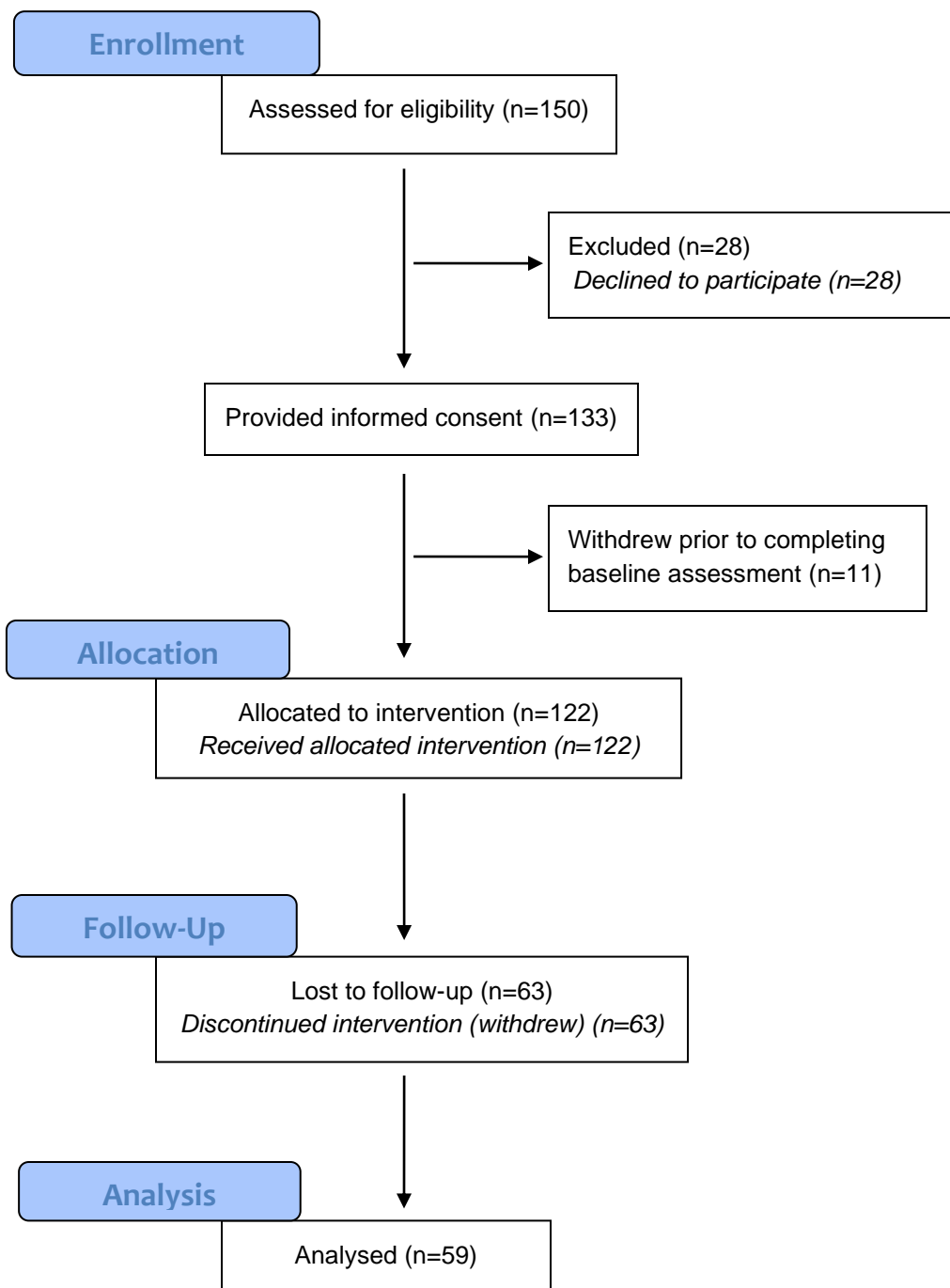

Supplement: Multimedia Appendix 12 [file formative_v5i9e28784_app12.pdf]
