# Supplementary material for: A Smartphone App to Support Adherence to Inhaled Corticosteroids in Young Adults With Asthma: Multi-Methods Feasibility Study
Source: JMIR Form Res. 2021 Sep 1;5(9):e28784. doi: 10.2196/28784 (PMC8444040; doi:10.2196/28784)
Supplement: Multimedia Appendix 13 [file formative_v5i9e28784_app13.pdf]

Table S2. Outcomes of Go- and No-Go progression criteria

	**Go – Proceed with RCT**	**Amend – Proceed with changes**	**Stop – Do not proceed unless changes are possible**
**1.Feasibility of participant recruitment**			
Can ≥74 participants be recruited to take part in the study?	*≥74 participants: 122 participants were recruited to take part in the study*	N/A[a]	N/A
**2. Feasibility of participant retention**			
Can ≥59 participants be retained in the study until completion?	*≥59 retained: 59/122 (48.4%) participants were retained in the study until completion*	N/A	N/A
**3. Usability of 'AsthmaMD'**			
Will the app receive a mean SUS score >68?	N/A	*SUS ≥63: AsthmaMD received a mean SUS score of 63.1 (SD=20.1)*	N/A
and			
Interpretation of qualitative data relating to usability	N/A	*App judged as usable*	N/A
**4. Acceptability of 'AsthmaMD'**			
Will the app receive a mean score ≥5 for overall user satisfaction?	*≥5 overall satisfaction: AsthmaMD received a mean score of 5.8 (SD=2.2)*	N/A	N/A
or			
Will ≥30% of participants agree to 3/5 acceptability-related questions?	*≥30% agree to 3/5 questions: >30% agreed/strongly agreed to 4/5 questions*	N/A	N/A
and			
Interpretation of qualitative data relating to acceptability	*App judged as highly acceptable*	N/A	N/A
**5. Feasibility of 'AsthmaMD'**			
Did ≥30% of participants use the app ≥1 day per week?	*≥30% used app ≥1 day/week: 83.1% used the app ≥1 day/week*	N/A	N/A
or			
Would ≥30% of participants continue to use the app after the study?	N/A	*≥25% continue to use app: 27.1% would continue to use the app*	N/A
and			
Interpretation of qualitative data relating to feasibility	*App judged as highly feasible*	N/A	N/A

[a]N/A: not applicable
